# Functional and Structural Characterization of OXA-935, a Novel OXA-10-Family β-Lactamase from Pseudomonas aeruginosa

**DOI:** 10.1128/aac.00985-22

**Published:** 2022-09-21

**Authors:** Nathan B. Pincus, Monica Rosas-Lemus, Samuel W. M. Gatesy, Hanna K. Bertucci, Joseph S. Brunzelle, George Minasov, Ludmilla A. Shuvalova, Marine Lebrun-Corbin, Karla J. F. Satchell, Egon A. Ozer, Alan R. Hauser, Kelly E. R. Bachta

**Affiliations:** a Department of Microbiology-Immunology, Northwestern University, Feinberg School of Medicine, Chicago, Illinois, USA; b Center for Structural Genomics of Infectious Diseases, Northwestern University, Feinberg School of Medicine, Chicago, Illinois, USA; c Department of Medicine, Division of Infectious Diseases, Northwestern University, Feinberg School of Medicine, Chicago, Illinois, USA; d Northwestern Synchrotron Research Center, Life Sciences Collaborative Access Team, Northwestern University, Argonne, Illinois, USA; e Center for Pathogen Genomics and Microbial Evolution, Institute for Global Health, Northwestern University, Feinberg School of Medicine, Chicago, Illinois, USA

**Keywords:** *Pseudomonas aeruginosa*, OXA-β-lactamase, antimicrobial resistance, ceftazidime, crystal structure

## Abstract

Resistance to antipseudomonal penicillins and cephalosporins is often driven by the overproduction of the intrinsic β-lactamase AmpC. However, OXA-10-family β-lactamases are a rich source of resistance in Pseudomonas aeruginosa. OXA β-lactamases have a propensity for mutation that leads to extended spectrum cephalosporinase and carbapenemase activity. In this study, we identified isolates from a subclade of the multidrug-resistant (MDR) high risk P. aeruginosa clonal complex CC446 with a resistance to ceftazidime. A genomic analysis revealed that these isolates harbored a plasmid containing a novel allele of *bla*_OXA-10_, named *bla*_OXA-935_, which was predicted to produce an OXA-10 variant with two amino acid substitutions: an aspartic acid instead of a glycine at position 157 and a serine instead of a phenylalanine at position 153. The G157D mutation, present in OXA-14, is associated with the resistance of P. aeruginosa to ceftazidime. Compared to OXA-14, OXA-935 showed increased catalytic efficiency for ceftazidime. The deletion of *bla*_OXA-935_ restored the sensitivity to ceftazidime, and susceptibility profiling of P. aeruginosa laboratory strains expressing *bla*_OXA-935_ revealed that OXA-935 conferred ceftazidime resistance. To better understand the impacts of the variant amino acids, we determined the crystal structures of OXA-14 and OXA-935. Compared to OXA-14, the F153S mutation in OXA-935 conferred increased flexibility in the omega (Ω) loop. Amino acid changes that confer extended spectrum cephalosporinase activity to OXA-10-family β-lactamases are concerning, given the rising reliance on novel β-lactam/β-lactamase inhibitor combinations, such as ceftolozane-tazobactam and ceftazidime-avibactam, to treat MDR P. aeruginosa infections.

## INTRODUCTION

Infections caused by multidrug-resistant (MDR) and extensively drug-resistant (XDR) organisms are an increasing threat to public health. Leading the way are infections by drug-resistant Gram-negative bacteria, such as Pseudomonas aeruginosa, Acinetobacter baumannii, and members of the *Enterobacteriaceae* family. Of particular interest is the human pathogen P. aeruginosa, which is responsible for diverse infections, including bacteremia and pneumonia as well as urinary tract, skin, and soft tissue infections. At baseline, P. aeruginosa harbors a large array of antimicrobial resistance mechanisms as part of its intrinsic resistome, including AmpC (a chromosomally encoded cephalosporinase), Pseudomonas derived cephalosporinases (PDCs), mutations in DNA gyrase and topoisomerase IV that lead to fluoroquinolone resistance, aminoglycoside inactivating enzymes, basal and inducible antibiotic efflux pumps (e.g., MexAB-OprM, MexCD-OrpJ, MexEF-OprN, and MexXY-OprM), and a downregulation of porins (such as OprD) that leads to carbapenem resistance (1, 2). P. aeruginosa frequently acquires exogenous genetic material in the form of plasmids that carry other mobile genetic elements (e.g., integrons, transposons, ICE elements) that can contain series of antimicrobial resistance genes (3, 4). One such element, the class 1 integron in1697, was recently identified as part of a novel antimicrobial resistance (AMR) plasmid discovered in sequence type (ST) 298*, an XDR subclade within the globally distributed P. aeruginosa high-risk clonal complex 446 (CC446) (3). The characterization of in1697 revealed the presence of several resistance gene cassettes, including genes for resistance to sulfonamides, quaternary ammonium compounds, and aminoglycosides. In most strains, in1697 also contained the β-lactamase gene, *bla_OXA-10_*.

OXA-10 (PSE-2) is a class D β-lactamase that was originally described in the late 1970s (5, 6) and confers resistance to cefotaxime and ceftriaxone but not to ceftazidime (7). Natural and laboratory-selected variants of OXA-10 have been identified, many of which differ in the spectrum of β-lactams that they hydrolyze (8). One such variant, OXA-14, contains a single amino acid change (glycine to aspartic acid at position 157, G157D) from the OXA-10 parent. The OXA-14 variant has been implicated in clinically significant resistance to ceftazidime (8, 9). In P. aeruginosa, variations in OXA-10-type enzymes are a rich source of expanding AMR, as evidenced by several recently identified members of the class that confer resistance to carbapenems: OXA-40, OXA-198, OXA-655, and OXA-656 (10–13).

Although the isolates within the ST298* collection of P. aeruginosa were typically susceptible to ceftazidime, we noted three isolates that were resistant (3). Interestingly, these three isolates harbored a derivative of in1697 in which the *bla_OXA-10_* gene contained two mutations that resulted in a glycine to aspartic acid substitution at position 157 (similar to OXA-14) and a phenylalanine to serine substitution at position 153. In the present study, we characterized this novel OXA-10 variant, which we designate “OXA-935”. We also determined the crystal structures of both OXA-14 and OXA-935. The structures of OXA-14 and OXA-935 shared most of the structural features previously described for OXA-10 (14); however, OXA-935 contained a second amino substitution, phenylalanine to serine at position 153 (F153S), that increased the flexibility of the Ω-loop, thereby resulting in the loss of carbamylation at the active site residue lysine 70 (K70) and in increased ceftazidime hydrolysis.

## RESULTS

### Identification of OXA-935, a novel class D, OXA-10 family β-lactamase.

In a recent study, we described a prolonged epidemic of ST298* XDR P. aeruginosa at a single academic center (3). Many of these isolates possessed sequence alignment to a novel AMR plasmid (pPABL048) harboring the integron in1697 (Fig. S1). This plasmid, originally described in the isolate PABL048, confers resistance to anti-pseudomonal penicillins, likely due to the presence of *bla*_OXA-10_ in the integron. We noted that three isolates (PS1793, PS1796, and PS1797) showed high levels of ceftazidime resistance not found elsewhere in this collection. Additionally, all three were nonsusceptibile to ceftazidime-avibactam, and PS1796 and PS1797 were nonsusceptible to ceftolozane-tazobactam. All three retained susceptibility to imipenem-relebactam (Table 1). These three isolates were closely related and were found to possess single nucleotide variants (SNVs) in the plasmid-borne *bla*_OXA-10_ gene, leading to G157D and F153S amino acid substitutions in OXA-10 and thereby yielding a novel variant allele now named OXA-935. We screened the genomes of all three isolates for additional changes in proteins implicated in β-lactam resistance (2). Most of the AMR proteins screened had sequences similar to the susceptible isolates PAO1 and PA14 (Table S1). Notably, PS1793, PS1796, and PS1797 contained a single nucleotide insertion in *ampD*, resulting in a frameshift leading to its inactivation. In our prior study (3), the same mutation was interpreted as a Δ2-30 amino acid deletion. This difference was the result of an alternate annotation method (prokka v1.12). The inactivation of AmpD leads to the cytoplasmic accumulation of cell wall fragments that bind to AmpR, leading to the de-repression of the chromosomally encoded cephalosporinase AmpC. The de-repression of AmpC is linked to increased resistance to cephalosporins (15–17). Mutations in AmpC have also been linked to extended spectrum β-lactam resistance; however, the AmpC allele (PDC-16) found in PS1793, PS1796, and PS1797 has not been associated with extended spectrum activity (18). We also identified a four amino acid deletion in NalC that was previously noted in the ceftazidime susceptible ST298* isolate PABL048 (3). NalC mutations have been linked with the increased expression of the MexAB-OprM efflux system and increased β-lactam resistance (19). In this study, we explored the molecular mechanism of ceftazidime resistance in PS1793, PS1796, and PS1797 with a specific focus on their OXA-10-variant β-lactamase, OXA-935.

**TABLE 1:** Minimal inhibitory concentrations (MICs) for parental and OXA935 deletion strains

	MICs (μg/mL) for *P. aeruginosa* strains					
Antibiotic	PS1793	PS1793 Δ*bla_oxa935_*	PS1796	PS1796 Δ*bla_oxa935_*	PS1797	PS1797 Δ*bla_oxa935_*
Aztreonam	16 (ns)^*b*^	16 (ns)	16 (ns)	16 (ns)	16 (ns)	16 (ns)
Cefepime	16 (ns)	8	16 (ns)	4	32 (ns)	4
Ceftazidime	64 (ns)	8	64 (ns)	8	64 (ns)	4
Ceftazidime-avibactam^*a*^	>32 (ns)	4	>32 (ns)	4	>32 (ns)	4
Ceftolozane-tazobactam^*a*^	4	1	8 (ns)	0.5	8 (ns)	1
Piperacillin-tazobactam^*a*^	64 (ns)	32 (ns)	64 (ns)	32 (ns)	128 (ns)	32 (ns)
Cefiderocol	0.25	<0.03	0.25	<0.03	0.25	<0.03
Meropenem	16 (ns)	16 (ns)	16 (ns)	8 (ns)	16 (ns)	8 (ns)
Imipenem	8 (ns)	8 (ns)	8 (ns)	8 (ns)	8 (ns)	8 (ns)
Imipenem-relebactam^*a*^	1	2	1	2	2	2
Ciprofloxacin	32 (ns)	32 (ns)	32 (ns)	16 (ns)	32 (ns)	16 (ns)
Gentamicin	>128 (ns)	>128 (ns)	>128 (ns)	>128 (ns)	>128 (ns)	>128 (ns)
Amikacin	32 (ns)	32 (ns)	32 (ns)	32 (ns)	32 (ns)	32 (ns)
Colistin	1	0.5	1	<0.25	1	<0.25

a Avibactam, tazobactam, and relebactam were present at a fixed dose of 4 μg/mL.

b ns, nonsusceptible (intermediate and resistant).

We performed long-read sequencing of PS1793 and used the results, along with previously generated Illumina short reads (3), to assemble a complete genome sequence for PS1793. This yielded a 7.4 Mb genome, consisting of a 6,868,713 bp circular chromosome and 3 circular plasmids. Surprisingly, the AMR plasmid pPABL048 described in our previous study aligned to two separate plasmid sequences in PS1793 (the 318,215 bp PS1793_p1 and the 113,189 bp PS1793_p2) with approximately 19 kb of overlapping sequence present on both plasmids (Fig. S2A–D). No additional sequence was present in these plasmids, suggesting that pPABL048 may, in fact, be a hybrid plasmid. The pPABL048 plasmid contains more than one set of replication and partitioning machinery, similar to other plasmids in the same family that possess both the IncP-2 system and the uncharacterized replication gene described in our previous study (3, 20). The AMR integron is present in the larger PS1793_p1 plasmid. PS1793 also harbors an additional 69,506 bp plasmid (PS1793_p3) that was not previously described in PABL048 (Fig. S2A–D). However, following a BLAST search, portions of this third plasmid aligned with the PABL048 chromosome (a 10 kb fragment aligned with 99% identity, and a 2.3 kb fragment aligned with 76% identity) and shared homology with other Pseudomonas genus plasmids. Based on alignments with the PS1793 complete genome, PS1796 was identical (with 0 chromosomal or plasmid SNVs), and PS1797 differed by a single chromosomal SNV (Table S2).

Comparison of the *bla*_OXA-10_ variant in PS1793, PS1796, and PS1797 to the National Center for Biotechnology Information (NCBI) database revealed that this allele had not been previously described. As such, it was assigned the name *bla*_OXA-935_ (RefSeq ID: WP_141989064.1). When its predicted protein sequence was compared to other protein sequences within the OXA-10 family, the G157D substitution was present in multiple homologs, including OXA-11 and OXA-14, which are known to confer extended spectrum resistance to ceftazidime (9, 21). The F153S substitution was unique to OXA-935, although OXA-795 possesses a deletion at positions 153 and 154 (Fig. S3). OXA-795 also confers ceftazidime resistance without an accompanying G157D substitution (22). Phylogenetic analysis of these OXA-10 family proteins showed that they are divided into two major groups and are classified by their first identified and earliest-named members: OXA-7 and OXA-10. OXA-935 belongs to the OXA-10 subgroup and is most closely related to OXA-11, OXA-14, OXA-16, and OXA-142, although most branches within each group have low bootstrap confidence, likely secondary to limited sequence variability (Fig. S2E).

We next sought to determine the prevalence of *bla*_OXA-10_ family genes within the Pseudomonas genus. By screening 9,799 genomes, we found that *bla*_OXA-10_-like genes were most common in P. aeruginosa (*n* = 196) but were also present in other species, including *P. stuzeri* (*n* = 8) and P. putida (*n* = 2) (Table S3). The *bla*_OXA-10_ allele was present in a diverse set of species and in P. aeruginosa STs, while other variants were more likely to be limited to a few or to a single ST. Thus far, *bla*_OXA-935_ has only been detected in the three ST298* P. aeruginosa isolates described in this study (Table S4).

### Expression of OXA-935 confers high level ceftazidime resistance.

We sought to characterize the role of the novel β-lactamase allele, *bla*_OXA-935,_ in ceftazidime resistance. Given that OXA-935 shared the G157D variation with OXA-14 and that the expression of OXA-14 has been previously linked to ceftazidime resistance (8, 9), we hypothesized that the novel β-lactamase allele *bla_OXA_*_-935_ conferred ceftazidime resistance in our clinical isolates. The AMR profile of PS1793, PS1796, and PS1797 was confirmed using the broth microdilution (BMD) method (Table 1). All three isolates demonstrated similarly high levels of resistance to ceftazidime with a minimum inhibitory concentration (MIC) of 64 μg/mL. The deletion of *bla*_OXA-935_ from the AMR plasmids of PS1793, PS1796, and PS1797 resulted in a reduction of the ceftazidime MICs to 8 μg/mL, 8 μg/mL, and 4 μg/mL, respectively, yielding susceptible phenotypes. The deletion of *bla*_OXA-935_ also made the strains susceptible to ceftazidime-avibactam and cefepime. Interestingly, *bla*_OXA-935_ deletion also resulted in a >8-fold reduction in the MIC of cefiderocol. Previously, when we deleted the entire pPABL048 plasmid from related strains in CC446, we detected the loss of resistance to piperacillin-tazobactam, which we did not observe with the single gene deletion (3). The deletion of *bla*_OXA-935_ resulted in a minor reduction of the piperacillin-tazobactam MIC (2 to 4-fold), but it did not impact the MIC of aztreonam. We did not observe any differences in susceptibility to meropenem or imipenem, suggesting that OXA-935 is unlikely to contribute to carbapenem resistance. Other than alterations in the MICs of cephalosporins, all three of the Δ*bla*_OXA-935_ deletion strains retained their resistance to gentamicin and ciprofloxacin and their susceptibility to colistin (Table 1). In pPABL048, *bla*_OXA-10_ is the third gene within the AMR integron in1697, and it has no discernible promoter immediately upstream. Therefore, it was unclear to what extent *bla*_OXA-935_ was expressed in PS1793, PS1796, and PS1797. To assess baseline expression and determine whether the expression was induced by ceftazidime, we performed qRT-PCR on all three isolates. The *bla*_OXA-935_ gene was expressed similarly to the control gene *rpoD*, and it was not induced by treatment with 0.5 MIC ceftazidime (Fig. S4A–C). Taken together, these results confirm that ceftazidime resistance in PS1793, PS1796, and PS1797 is largely driven by the presence and expression of *bla*_OXA-935_.

To evaluate the impact of the G157D variant of OXA-14 and the G157D and F153S variants of OXA-935 on β-lactam resistance, *bla*_OXA-10,_ *bla*_OXA-14,_ and *bla*_OXA-935_ were individually cloned into plasmid pPSV37 under the control of the isopropyl β-D-1-thiogalactopyranoside (IPTG) inducible lacUV5 promoter and expressed in P. aeruginosa PAO1 and PA14. The inducible expression of OXA-935 was confirmed by qRT-PCR (Fig. S5). MIC data for PAO1 and PA14 expressing OXA-10, OXA-14, and OXA-935 are shown in Table 2. Compared to strains expressing the pPSV37 vector control, the expression of OXA-14 resulted in a 16-fold increase in the ceftazidime MIC and an 8-fold increase in the ceftazidime-avibactam MIC in PAO1 and a 16-fold increase in the MICs of both antibiotics in PA14, consistent with previous reports (8, 9, 22). The expression of OXA-935 resulted in a 32-fold increase in the ceftazidime and ceftazidime-avibactam MICs in PAO1 and 32-fold and 8-fold increases in the ceftazidime and ceftazidime-avibactam MICs in PA14, respectively. These findings provide evidence that both OXA-14 and OXA-935 confer resistance to ceftazidime and ceftazidime-avibactam but that the expression of OXA-935 has, in general, a greater impact on the MICs. Increasing the ITPG induction of OXA-14 and OXA-935 revealed a dose-response relationship between β-lactamase expression and the ceftazidime MIC in both PAO1 and PA14, with OXA-935 expression yielding a maximum MIC of 32 μg/mL (Table 3). In addition, the expression of OXA-14 resulted in a 4-fold increase in the cefiderocol MIC in both PAO1 and PA14, resulting in nonsusceptibility for PA14 expressing OXA-14. The expression of OXA-14 resulted in a 4-fold increase in the piperacillin-tazobactam MIC in both strains, while the expression of OXA-935 resulted in no increase. The impact on cefepime susceptibility was more uniform in that the expression of all three proteins, OXA-10, OXA-14, and OXA-935, resulted in a 4-fold increase in the MIC when expressed in either strain background. These results were consistent with previous findings, indicating that OXA-10 has activity against piperacillin-tazobactam but not against ceftazidime and mirrored findings previously published on ceftazidime resistance and OXA-14 (8, 14). Surprisingly, OXA-14 also increased cefiderocol resistance. In contrast, OXA-935 did not provide resistance to piperacillin-tazobactam in these isolate backgrounds, but it did confer substantial resistance to ceftazidime and ceftazidime-avibactam.

**TABLE 2:** MICs for PAO1 and PA14 induced to express OXA-10, OXA-14, and OXA-935

		MICs (μg/mL)									
Strain^*a*^	aa differences	ATM^*b*^	FEP	CAZ	CZA^*c*^	C/T	TZP^*c*^	FDC	MEM	IPM	I-R^*c*^
PAO1 + pPSV37	NA	4	1	1	1	0.25	2	<0.03	0.5	<2	0.25
PAO1 + pPSV37-*oxa10*		8	4	1	1	0.5	16	<0.03	1	<2	0.5
PAO1 + pPSV37-*oxa14*	G157D	8	4	16 (ns)^*d*^	8	2	8	0.12	0.5	<2	0.25
PAO1 + pPSV37-*oxa935*	F153S, G157D	4	4	32 (ns)	32 (ns)	4	2	0.12	0.5	<2	0.25
PA14 + pPSV37	NA	4	1	1	2	0.5	2	2	0.5	<2	0.5
PA14 + pPSV37-*oxa10*		8	4	1	2	0.5	32 (ns)	4	0.5	<2	0.5
PA14 + pPSV37-*oxa14*	G157D	8	4	16 (ns)	16 (ns)	2	8	8 (ns)	<0.25	<2	0.5
PA14 + pPSV37-*oxa935*	F153S, G157D	8	4	32 (ns)	16 (ns)	2	2	4	<0.25	<2	0.5

a All strains were induced with 1 mM IPTG to drive the expression of various OXA proteins.

b ATM, aztreonam; FEP, cefepime; CAZ, ceftazidime; CZA, ceftazidime-avibactam; C/T, ceftolozane-tazobactam; TZP, piperacillin-tazobactam; MEM, meropenem; IPM, imipenem; I-R, imipenem-relebactam; FDC, cefiderocol.

c Avibactam, tazobactam and relebactam were present at a fixed dose of 4 μg/mL.

d (ns), nonsusceptible (intermediate and resistant).

**TABLE 3:** OXA-14 and OXA-935 induction increase ceftazidime resistance in PAO1 and PA14

	Ceftazidime MICs (μg/mL)				
mM IPTG	0	0.25	0.5	0.75	1
PAO1 + pPSV37	1	1	1	1	1
PAO1 + pPSV37-*oxa10*	1	1	1	1	1
PAO1 + pPSV37-*oxa14*	1	8	8	16 (ns)	16 (ns)
PAO1 + pPSV37-*oxa935*	2	16 (ns)^*a*^	16 (ns)	32 (ns)	32 (ns)
PA14 + pPSV37	1	1	1	1	1
PA14 + pPSV37-*oxa10*	1	1	1	1	1
PA14 + pPSV37-*oxa14*	4	8	16 (ns)	16 (ns)	16 (ns)
PA14 + pPSV37-*oxa935*	8	16 (ns)	32 (ns)	32 (ns)	32 (ns)

a (ns), nonsusceptible (intermediate and resistant).

### The amino acid change F153S introduced high flexibility into the Ω-loop of OXA-935.

**FIG 1:**
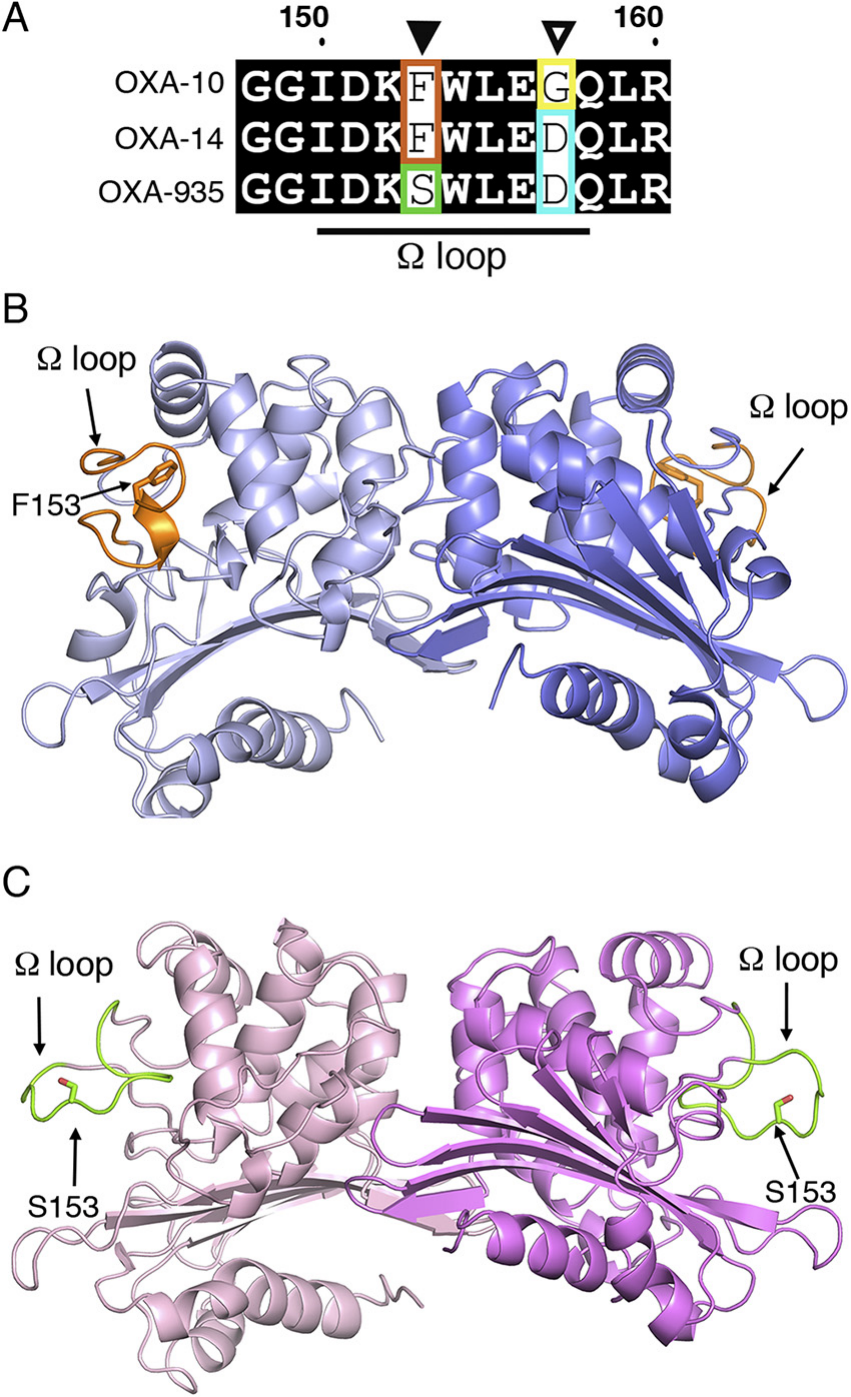
Genomic organization and structures of OXA-935 and OXA-14. (A) Sequence alignment of the Ω-loop of OXA-10, OXA-14, and OXA-935, indicating in green the change in the residue 153 (F→S) and in cyan the change in residue 157 (G→D). Cartoon representations of the asymmetric dimeric structures of (B) OXA-14 and (C) OXA-935, showing the Ω-loop in orange for OXA-14 and in green for OXA-935.

**FIG 2:**
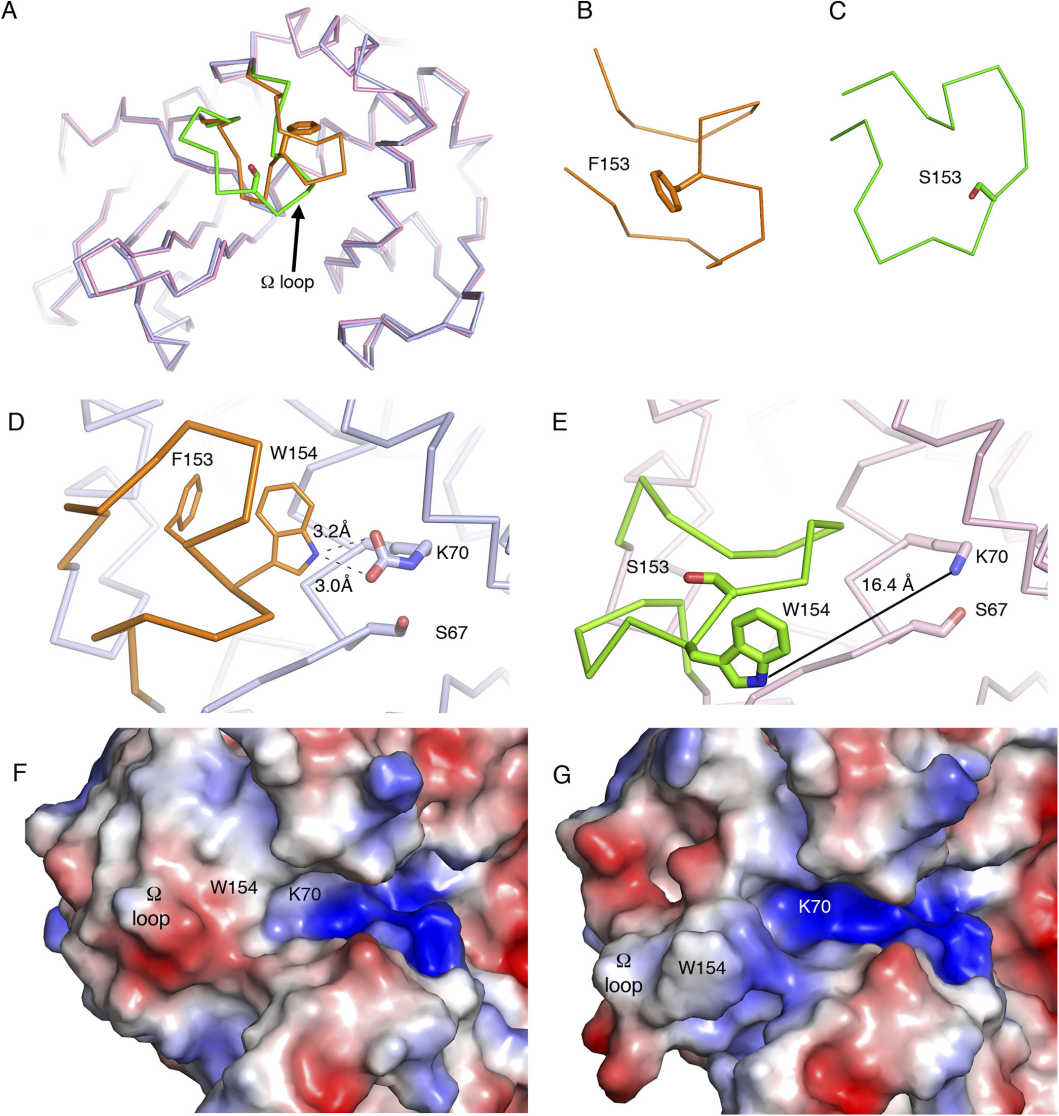
The F153S substitution disrupts the interactions of W154 in the Ω-loop of OXA-935 with the catalytic residue K70. (A) Structural alignment of OXA-14 (blue) and OXA-935 (pink), highlighting the Ω-loops in orange and green, respectively. Zoomed in view of the Ω-loops of (B) OXA-14 and (C) OXA-935. Positions of the Ω-loops and interactions of W154 and the catalytic residue K70 in (D) OXA-14 and (E) OXA-935. Dashed lines represent hydrogen bond interactions. The continuous black line shows the distance between K70 and W154 in OXA-935. Surface charge representation of the Ω-loops and the active sites of (F) OXA-14 and (G) OXA-935.

OXA-935 differs from OXA-14 in a single amino acid substitution in the protein’s Ω-loop at position 153 (Fig. 1A). Changes in this loop in multiple classes of β-lactamases are known to increase its flexibility and therefore increase substrate access to the active site (14, 18, 23). This may explain its increased activity against substrates with bulky side chains, such as ceftazidime. Since structures of OXA-14 and OXA-935 were unavailable to corroborate this hypothesis, we determined the crystal structures of both OXA-14 and OXA-935. We were able to determine apo structures for both enzymes (Fig. 1; Table S5) but not in complex with ceftazidime. Both protein structures contained two chains in the asymmetric unit, corresponding to a dimer, as previously described for OXA-10 (14, 24). OXA-935 (PDB code: 7L5V) belonged to the space group P2_1,_ and OXA-14 (PDB code: 7L5R) belonged to the space group P2_1_2_1_2_1_ (Tables S6 and S7). The structural alignment of these revealed a root mean square deviation of 0.65, driven primarily by the differences in the conformation of the Ω-loop, where the F153S mutation was located (Fig. 2A–C; Fig. S7). In chain A of OXA-14, the Ω-loop was closer to the active site, and the indole group from tryptophan 154 (W154) interacted with the carbamylated lysine 70 (K70) (3.2 Å), as was described in OXA-10 (14) (Fig. 2D). The interaction between W154 and K70 is critical for both the activity and the stability of OXA-10 (25). In chain B of OXA-14, K70 was decarbamylated, causing an open confirmation, consistent with what was observed for the heterodimeric structure of OXA-10 (14). In contrast, the Ω-loops of both chains in the crystal structure of OXA-935 had open conformations where the hydrogen bond between K70 and the indole group of W154 was absent (16.4 Å) and both of the K70 residues were decarbamylated (Fig. 2E; Fig. S7). This more open confirmation of the Ω-loop created a larger and more positively charged active site cavity, which may allow it to accommodate bulkier and more negatively charged substrates, such as ceftazidime (Fig. 2F and G). An additional structure of OXA-935 (PDB code: 7N1M) that was determined from different crystallization conditions and a different space group, P2_1_2_1_2_1_, also revealed that both of the monomers of OXA-935 had decarbamylated K70 residues and disordered Ω-loops, supporting the observations that S153 conferred significant flexibility to the Ω-loop (Tables S5–S7).

### OXA-935 hydrolyzes ceftazidime and cefepime *in vitro*.

**FIG 3:**
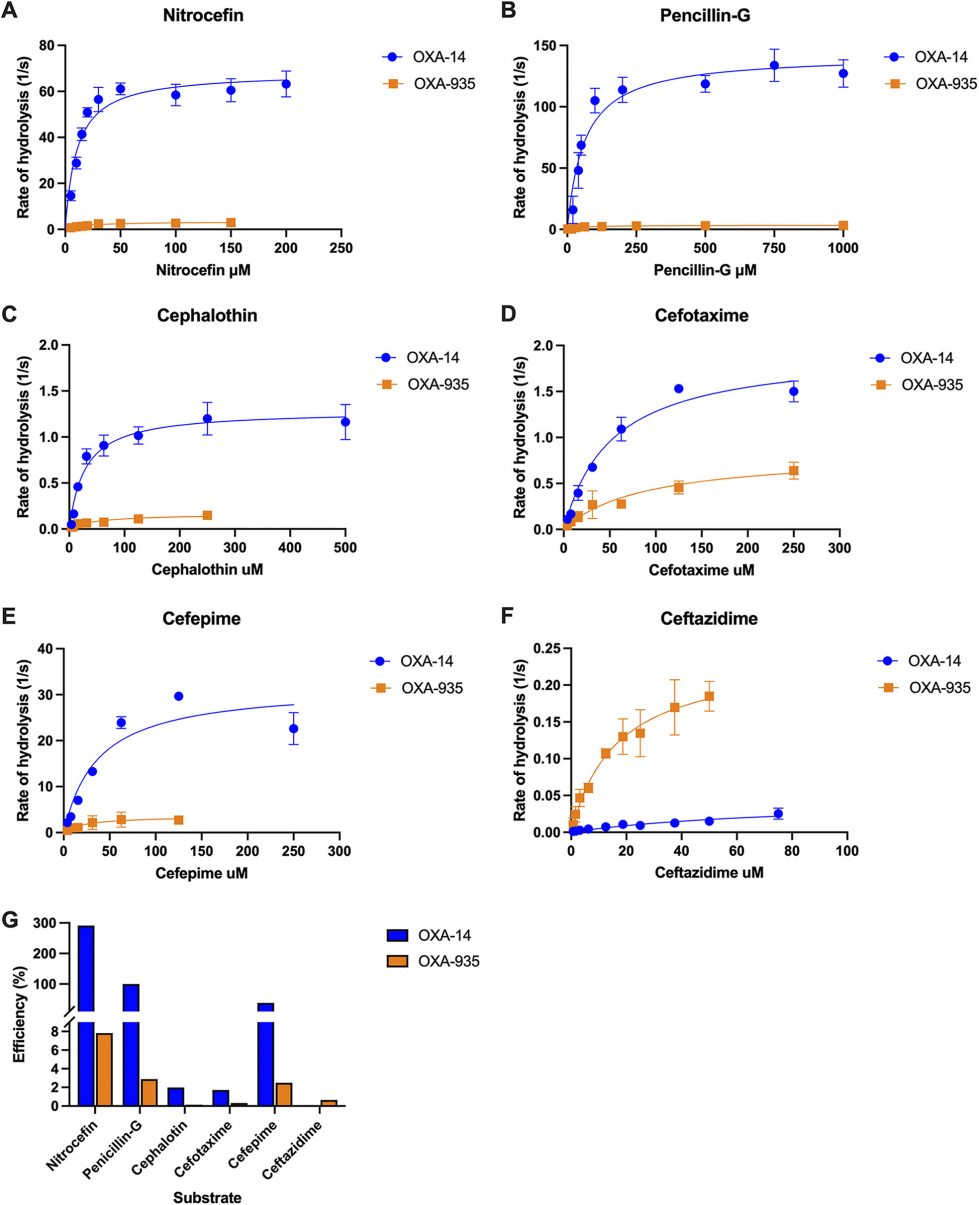
Hydrolysis of various β-lactam compounds by OXA-14 and OXA-935. Using purified OXA-14 (blue, circles) or OXA-935 (orange, squares), we measured the cleavage of β-lactam compounds in a saturated sodium bicarbonate solution, including nitrocefin (A), penicillin-G (B), cephalothin (C), cefotaxime (D), cefepime (E), and ceftazidime (F). Hydrolysis of each compound (1/s) was plotted versus the concentration of substrate and analyzed via Michaelis-Menten nonlinear analysis. Each enzyme-drug concentration was assayed in at least triplicate, and the data are presented as the mean (points) and standard deviation (bars). (G) The catalytic efficiency (*k*_cat_/*K*_m_) of every enzyme-drug pair is plotted as a percentage of the catalytic efficiency of pencillin-G cleavage by OXA-14 when normalized to 100%.

Variations in the Ω-loop have been shown to broaden the spectrum of β-lactamases. In OXA-935, the F153S variation conferred high flexibility to the Ω-loop, and this, in conjunction with a decarbamylated K70, might be deleterious to OXA-935 activity *in vitro*. Thus, we examined the rates of hydrolysis of a variety of β-lactam compounds by both OXA-935 and OXA-14, using saturated bicarbonate conditions that favor carbamylation (Fig. 3A–F; Table 4). First, we tested the hydrolysis of nitrocefin, a chromogenic cephalosporin, and observed that OXA-14 hydrolyzed nitrocefin more efficiently than did OXA-935 (Fig. 3A). We then determined the kinetic constants *K*_m_ and *k*_cat_ as well as the catalytic efficiency (*k*_cat_/*K*_m_) of OXA-14 and OXA-935 for various β-lactam antibiotics and normalized the efficiency of each enzyme-drug pair by assigning the efficiency of OXA-14 penicillin-G hydrolysis to 100% (Fig. 3G). Overall, OXA-14 hydrolyzed most of the tested β-lactams more efficiently than did OXA-935, specifically penicillin-G, cefepime, cephalothin, and cefotaxime (Fig. 3; Table 4). Both enzymes hydrolyzed cefepime efficiently, but the substrate affinity (OXA-14 *K*_m_ = 37 μM, OXA-935 *K*_m_ = 78 μM) was higher and the turnover rate (OXA-14 *k*_cat_ = 32 s^−1^, OXA-935 *k*_cat_ = 4.4 s^−1^) faster for OXA-14.

**TABLE 4:** Kinetic parameters of purified β-lactamases OXA-14 and OXA-935^*a*^

	OXA-14 (G157D)			OXA-935 (G157D, F153S)			*k_cat_*/*K_m_* ratio for OXA-935/OXA-14
Substrate	*K_m_* (μM) ± SD^*b*^	*k_cat_* (s^−1^) ± SD^*c*^	*k_cat_*/*K_m_* (μM^−1^ s^−1^)^*d*^	*K_m_* (μM) ± SD	*k_cat_* (s^−1^) ± SD	*k_cat_*/*K_m_* (μM^−1^ s^−1^)	
Nitrocefin	11 ± 1	68 ± 1	6.2	19 ± 3	3.4 ± 0.3	0.18	0.029
Penicillin G	64 ± 16	144 ± 11	2.2	52 ± 10	3.4 ± 0.4	0.065	0.030
Cephalothin	29 ± 7	1.3 ± 0.2	0.045	49 ± 7	0.16 ± 0.01	0.0033	0.073
Cefotaxime	50 ± 8	1.9 ± 0.2	0.038	123 ± 78	0.90 ± 0.13	0.0073	0.19
Cefepime	37 ± 3	32 ± 3	0.86	78 ± 30	4.4 ± 0.8	0.056	0.065
Ceftazidime	51 ± 18	0.034 ± 0.014	0.00067	17 ± 1	0.24 ± 0.02	0.014	21
Meropenem	>250	ND^*e*^	ND	>250	ND	ND	ND
Imipenem	>250	ND	ND	>250	ND	ND	ND

a Data represent the mean ± standard deviation (SD) from three independent experiments.

b *K_m_*, Michaelis constant (substrate affinity).

c *k_cat_*, turnover rate.

d *k_cat_/K_m_*, specificity constant (catalytic efficiency).

e ND, not determinable due to a low initial rate of hydrolysis.

In contrast, OXA-935 showed higher affinity (*K*_m_ = 17 μM) and substrate turnover (*k*_cat_ = 0.24 s^−1^) for ceftazidime than did OXA-14. In fact, OXA-14 did not reach steady-state hydrolysis for ceftazidime, even at the highest concentration for which hydrolysis was detectable within a linear range (Fig. 3F; Table 4). Finally, although the hydrolysis of carbapenems (meropenem and imipenem) was tested, the rate of hydrolysis detected under the assay conditions did not reach steady-state, and the *K*_m_ values of both were reported as >250 μM (Table 4), confirming that neither enzyme functions well as a carbapenemase. Therefore, OXA-935, despite having a more flexible Ω-loop and decarbamylated K70, hydrolyzed ceftazidime more efficiently *in vitro* than did OXA-14, consistent with the observed ceftazidime resistance phenotype in our P. aeruginosa isolates.

The carbamylation of K70 in other class D β-lactamases is favored at a more basic pH (26, 27), whereas OXA-935 showed decarbamylated K70 even at a pH of 8.3. We hypothesized that the F153S substitution conferred enough flexibility to the Ω-loop to destabilize the carbamylated state of K70, even at a basic pH. To corroborate this hypothesis, we used nitrocefin hydrolysis as a reporter to examine the activity of OXA-14 and OXA-935 across a range of pH values (pH 7.0 to 8.5) in a saturated sodium bicarbonate buffer. Sodium bicarbonate stimulated the activity of both enzymes across the tested pH range (Fig. S6). We observed an increase in nitrocefin hydrolysis for OXA-14 as the pH value increased, suggesting that K70 was more stably carbamylated at a higher pH. Conversely, we observed little increase from the baseline in nitrocefin hydrolysis by OXA-935 as the pH increased. Thus, unlike OXA-10, OXA-14, and other related β-lactamases, it is likely that the K70 of OXA-935 remains decarbamylated *in vitro*, despite increases in pH (Fig. S6).

## DISCUSSION

In this study, we characterized a novel OXA-10-family β-lactamase, OXA-935, found in three XDR ST298* P. aeruginosa isolates that are unique with respect to their resistance to ceftazidime. OXA-935 differs from OXA-10 by two amino acid substitutions: G157D, which it shares with OXA-14, and F153S which is novel. We showed that the deletion of *bla*_OXA-935_ from our three isolates restored susceptibility to ceftazidime and that the expression of *bla*_OXA-935_ in laboratory P. aeruginosa strains was sufficient for resistance to ceftazidime. We also determined the crystal structures of both OXA-14 and OXA-935. In the crystal structure of OXA-935, the critical active site K70 residue was decarbamylated in both monomers, and the F153S substitution resulted in significant flexibility of the Ω-loop of OXA-935. These structural changes likely impacted the ability of OXA-935 to hydrolyze bulkier substrates, such as ceftazidime.

The most well-characterized pathway of AMR to cephalosporins, such as ceftazidime and the newer β-lactam/β-lactamase combination antimicrobials, is the accumulation of chromosomal mutations leading to the overproduction of the intrinsic cephalosporinase AmpC or mutations in AmpC that extend its spectrum of activity (18, 28). The overexpression of AmpC is often secondary to mutations in AmpD. AmpD inactivation in P. aeruginosa results in the de-repression of AmpC (16) and is known to lead to increased resistance. Sequencing of our three isolates revealed a frameshift in AmpD that resulted in a premature stop codon. This mutation was unique to our three isolates within the ST298* subclade and may explain why the ceftazidime MICs remained slightly elevated at 4 and 8 μg/mL in PS1793, PS1796, and PS1797 Δ*bla*_OXA-935_, respectively. The AmpC allele (PDC-16) found in our three isolates has not previously been associated with extended spectrum β-lactam resistance and is consistent across all isolates within the ST298* subclade (18). Despite the underlying inactivation of AmpD, the deletion of *bla*_OXA-935_ ultimately restored ceftazidime susceptibility, and its ectopic expression produced considerable resistance in laboratory strains of P. aeruginosa. This suggests that the ceftazidime and ceftazidime-avibactam resistance phenotypes observed in our isolates were largely driven by OXA-935 expression and were less impacted by the coexisting AmpD inactivation. Interestingly, the G157D and F153S substitutions had a more unpredictable impact on the MICs of additional β-lactam compounds. Compared to OXA-10, the expression of OXA-935 resulted in little change in the MICs of aztreonam and piperacillin-tazobactam and moderate increases in the MICs of cefepime, ceftolozane-tazobactam, and cefiderocol. The expression of OXA-10, OXA-14, and OXA-935 had little impact on carbapenem MICs, suggesting that these variants, unlike other enzymes in this class, do not harbor carbapenemase activity. These two amino acid substitutions, while extending the spectrum of activity against ceftazidime and ceftazidime-avibactam, had little impact on the limited ability of OXA-935 to hydrolyze other β-lactam compounds. Thus, there may be a trade-off to mutations that affect the confirmation of the active site and the flexibility of the Ω-loop.

In P. aeruginosa, there is a growing concern for the increasing spectrum of AMR among OXA-10-family β-lactamases. OXA-10 has been subjected to significant mutational pressure, both in the laboratory where ceftazidime (8), carbapenem (12, 13, 29), ceftolozane-tazobactam, and ceftazidime-avibactam (22, 30, 31) resistant mutants have been selected, and clinically, as evidenced by the sheer diversity of described mutations leading to clinically significant AMR (9, 21, 32–34). Class D β-lactamases share the same general structure with conserved motifs, including an active-site serine 67, a carbamylated lysine (K70), and a stabilizing and invariant tryptophan residue (W154) within the Ω-loop (Fig. S7, S8) (25). To better understand the impact of the OXA-935 amino acid substitutions on enzymatic function, we determined the crystal structures of OXA-14 and OXA-935. OXA-14 crystallized as a heterodimer in which, much like OXA-10, only one of the two K70 residues was carbamylated (26, 27). The Ω-loop conformation was maintained, and the hydrogen bond between the indole group of W154 and K70 was present. The F153S variant found within OXA-935 yielded a distinctly different structure of the Ω-loop, in which W154 was considerably more flexible and oriented away from the active site cavity, the carbamylation of K70 was not observed, and the active site groove was more positively charged. W154, in addition to stabilizing K70, helps orient K70 such that it has optimal interactions with other residues in the active site (25). Critically, mutations in W154 (W154C), the deletion of F153-W154, and duplications of negatively charged residues in this region have been identified in P. aeruginosa clinical isolates that are resistant to ceftazidime-avibactam and ceftolozane-tazobactam (22, 35), suggesting that these residues are important for modified activity against some of the newest β-lactam/β-lactamase combination antibiotics. Both F153 and its neighboring residue W154 may be critical sites of mutation that allow for increased substrate accessibility of the active site for ceftazidime, ceftazidime-avibactam, and ceftolozane-tazobactam.

In assessing functional kinetics, purified OXA-935 hydrolyzed ceftazidime *in vitro* more efficiently than did its parental enzyme OXA-14. These data were consistent with MIC testing, which revealed that the expression of OXA-935 was sufficient for greater ceftazidime resistance. The low *in vitro* rate of hydrolysis of OXA-935 compared to that of OXA-14 for most substrates could be related to the decarbamylation of K70, as seen in the crystal structure. Attempts to crystalize OXA-14 or OXA-935 with bound ceftazidime were unsuccessful. Regardless, the new structures of OXA-14 and OXA-935 provide structural insights into the interplay between the Ω-loop and the active site, which governs the broad spectrum of activity for this family of enzymes.

Finally, OXA-10-family β-lactamases are often found in mobile transmissible elements, such as plasmids (22). OXA-935 is no exception in that it is located within a plasmid-borne integron that harbors additional AMR elements for aminoglycosides, quaternary ammonium compounds, and sulfonamide resistance (3). The mobility of OXA-10-family β-lactamases, coupled with their propensity for mutation in the face of antibiotic pressure, makes this enzymatic class one of growing concern. The discovery of yet another variant of OXA-10, namely, OXA-935, that confers ceftazidime and ceftazidime-avibactam resistance as well as decreased susceptibility to cefepime and ceftolozane-tazobactam prior to the clinical introduction of both combination agents is worrisome. Minimal amino acid changes in OXA-10 lead to clinically significant extended spectrum β-lactamase and carbapenemase activity, especially when located in the Ω-loop. Most concerning is the development of resistance to ceftolozane-tazobactam and ceftazidime-avibactam, which are used as last-resort therapies to treat MDR P. aeruginosa infections. Ultimately, our findings emphasize the importance of continued molecular surveillance of MDR P. aeruginosa and of increased recognition of the contribution of mobile OXA-10-family β-lactamases to AMR phenotypes.

## MATERIALS AND METHODS

### Bacterial strains and growth conditions.

P. aeruginosa PS1793, PS1796, and PS1797 are clinical strains from the respiratory tracts of patients at Northwestern Memorial Hospital (NMH), isolated between 2005 and 2007, and PABL048 is a clinical strain from the bloodstream of a patient at NMH, isolated in 2001 (3). PA14 and PAO1 are commonly used laboratory strains (36, 37). Relevant characteristics of these strains are listed in Table S8.

Escherichia coli strain TOP-10 (Invitrogen) was used for cloning, and E. coli strains S17.1 λpir (38) and SM10 λpir were used to introduce plasmids into P. aeruginosa. E. coli BL21(DE3) with and without the pMagic plasmid (39) was used for protein expression. Bacterial strains were streaked from frozen cultures onto LB agar and, unless otherwise stated, grown at 37°C in LB.

Antibiotics were used at the following concentrations: irgasan 5 μg/mL (irg), hygromycin 500 μg/mL (hyg), and gentamicin 100 μg/mL (gent) for P. aeruginosa; gentamicin 15 μg/mL, hygromycin 100 μg/mL, kanamycin 50 μg/mL (kan), and ampicillin 200 μg/mL (amp) for E. coli. Further details on the strains and plasmids used in this study can be found in Tables S8 and S9.

### Hybrid assembly of PS1793 and comparison to PABL048.

For short-read and long-read sequencing, genomic DNA was extracted from an overnight culture of PS1793 using a Promega Maxwell Cell DNA Purification Kit (Promega Corp., Madison, WI). For short-read sequencing, a sequencing library was prepared using a Nextera XT Kit (Illumina, San Diego, CA) and sequenced using an Illumina MiSeq instrument and a v3 flow cell, yielding 2 × 301 bp paired-end reads for a total of 1,174 Mbp of sequence with an approximate coverage of 160-fold. For long-read sequencing, genomic DNA from PS1793 was used to create a sequencing library using ligation sequencing kit SQK-LSK109 (Oxford Nanopore, United Kingdom, catalog number: SQK-LSK109) and sequenced on the Oxford Nanopore MinION platform, using a FLO-MIN106 flow cell. Base calling with default quality score filtering and demultiplexing of sequenced reads was performed using Guppy (v3.4.5), yielding 31,050 reads for a total of 297 Mbp of sequence and an approximate coverage of 41-fold. Hybrid genome assembly was performed using Unicyler (v0.4.8) (40) with the default settings to generate a single, circular 6,868,713 bp chromosome and 3 circular plasmids totaling 318,215 bp, 113,189 bp, and 69,506 bp, respectively. The PS1793 complete genome was annotated using the NCBI Prokaryotic Genome Annotation Pipeline (PGAP) (v5.2) (41, 42) and is available through NCBI BioSample SAMN12162657 and GenBank locus accessions CP083366 to CP083369. PS1793 plasmid contigs and pPABL048 were aligned to each other and visualized using BRIG (v0.95) (43).

### Sequencing of PS1796 and PS1797 and comparison to PS1793.

For the short-read sequencing of PS1796 and PS1797, genomic DNA was extracted from an overnight culture as described previously. Sequencing libraries were prepared using a Nextera XT Kit (Illumina, San Diego, CA) and sequenced using an Illumina MiSeq instrument and v3 flow cell as described previously. Reads were quality trimmed, and adapter sequences were removed using Trimmomatic (v0.36) (44). The trimmed reads were aligned to the PS1793 complete genome (chromosome and plasmids) using BWA (v0.7.15) (45) (https://arxiv.org/abs/1303.3997v2) and sorted and indexed using samtools (v0.1.19-44428cd) (46). SNV sites were then identified as described previously (3).

### Alignment and phylogenetic comparison of OXA-10-family β-lactamases.

OXA-10 family β-lactamase sequences were obtained from the NCBI Pathogen Detection Reference Gene Catalog using “OXA-10” as the search query in January of 2021. OXA-16 was also included, based on a literature review (32). For alignment visualization, multiple alignment of all OXA-10 family β-lactamase amino acid sequences was performed using Qiagen CLC Sequence Viewer (v8.0) with the default parameters. For the phylogenetic analysis, we used all OXA-10 family β-lactamase amino acid sequences and included OXA-5, a relatively closely related class D β-lactamase, as an outgroup protein (47). The Sequences were aligned using MUSCLE (v3.8.31) (48). A maximum likelihood phylogenetic tree was constructed based on this alignment using RAxML (v8.2.11) (49) with automatic protein model selection, a gamma model of rate heterogeneity (-m PROTGAMMAAUTO), and 1,000 rapid bootstraps to assess support (-f a -N 1,000). The resulting tree was plotted using iTOL v.6 (50).

### Genus-wide screen for OXA-10-family β-lactamase prevalence.

Pseudomonas genus genomes available in the Pseudomonas Genome Database (51), along with the accompanying metadata (species, MLST) were obtained on November 10th, 2020. After the exclusion of one genome which failed to download, this yielded a total of 9,799 genomes (including both complete and draft genomes). Nucleotide BLAST alignments were performed using each OXA-10 family β-lactamase as the query sequence and each genome as the subject sequence. To identify which genomes contained known OXA-10 family β-lactamase genes, the BLAST results were parsed to identify alignments with 100% sequence identity and coverage. To identify genomes containing any OXA-10 family β-lactamase gene, the BLAST results were parsed to identify alignments with 90% sequence identity and coverage for *bla*_OXA-10_.

### Mutational resistance analysis.

To examine the role of mutational resistance, specifically to ceftazidime and other β-lactam compounds, a curated panel of genes from the NCBI Prokaryotic Genome Annotation Pipeline (PGAP) annotation of PS1793 was screened for mutations known to confer resistance in P. aeruginosa (1, 2, 18, 52). In cases where resistance is imparted by gain-of-function mutations, translated coding sequences were screened for previously reported alleles that are known to be involved in resistance (1, 2, 18, 53). In cases where resistance is conferred from loss-of-function mutations (e.g., gene disruption), translated coding sequences were compared to those of PAO1 and PA14 as reference genomes to assess gross changes in amino acid sequences (51). Multiple sequence alignment was performed using BLAST and Clustal Omega (https://www.ebi.ac.uk/Tools/common/tools/help/index.html?tool=clustalo).

### Generation of pEX18HygB and the *bla*_OXA-14_ and *bla*_OXA-935_ expression plasmids.

The hygromycin B resistance gene (*hygR*) and the associated promoter (*ampR* promoter) were amplified from the pFLP_hyg (54) plasmid using primers TT115 and TT116, thereby creating a 1,185 bp amplicon. Inverse PCR with primers TT113 and TT114 was used to amplify the backbone of the pEX18Ap plasmid (55), excluding the ampicillin resistance gene and the associated promoter, resulting in a 4,644 bp amplicon. Both amplicons were designed with matching overhangs to allow for annealing via Gibson assembly. The *hygR* insert and the inverse PCR product of the pEX18Ap backbone were mixed at a 5:1 ratio and ligated for 30 min at 50°C using a New England Biolabs (NEB) Gibson Assembly Cloning Kit to create pEX18HygB. The resulting assembled product was transformed into chemically competent E. coli TOP10 and plated on LB agar plates supplemented with 100 μg/mL hygromycin B (GoldBio, USA). An individual colony was picked, transferred to 5 mL of LB medium supplemented with 100 μg/mL hygromycin B, and incubated overnight at 37°C. After 14 h, pEX18HygB was isolated from E. coli TOP10 using a QIAprep Spin Miniprep Kit (Qiagen, Germany) and it’s sequence verified (using 9 pairs of overlapping primers, TT117 to TT125) at the NuSeq facility at Northwestern University. The resulting sequences confirmed the creation of the 5,829 bp plasmid pEX18HygB.

For the creation of OXA β-lactamase expression vectors for protein purification, both full-length (FL) and mature sequence (trunc, Δaa1-20) *bla*_OXA-14_ (NCBI Reference Sequence: WP_064056056.1) and *bla*_OXA-935_ (NCBI Reference Sequence: WP_141989064.1) were codon optimized (SmartGene) for expression in E. coli, synthesized (Twist Bioscience), and cloned into the pMCSG53 vector (56), which contains a tobacco etch virus (TEV) cleavable N-terminal 6× His tag, ampicillin resistance, and genes for rare codons.

For the creation of plasmids with isopropyl β-d-1-thiogalactopyranoside (IPTG)-inducible expression of *bla*_OXA-10_, *bla*_OXA-14_, and *bla*_OXA-935_, the full-length sequences (without their native promoters, given their location in the middle of an integron) were amplified from PABL048 (for *bla*_OXA-10_), pMCSG53-*oxa14* FL (for *bla*_OXA-14_), and PS1793 (for *bla*_OXA-935_), using the primers pPSV37_OXA_F_Gibs and pPSV37_OXA_R_Gibs, respectively. HindIII-digested medium-copy number plasmid pPSV37 (57) was mixed at a 1:3 ratio with each of the OXA β-lactamase gene products and ligated using the NEB Gibson Assembly Cloning Kit. The resulting vectors, pPSV37-*oxa10*, pPSV37-*oxa14*, and pPSV37-*oxa935*, were verified by sequencing using primers SeqFwPr and SeqRevPr PSV37, and they, along with a pPSV37 vector control, were transformed into electrocompetent (58) PAO1 and PA14. All of the recombinant methods, including the introduction of plasmids expressing OXA-10, OXA-14, and OXA-935 in PAO1 and PA14 were reviewed and approved by the Northwestern University Institutional Biosafety Committee. All primer sequences are listed in Table S9.

### Generation of Δ*bla*_OXA-935_ P. aeruginosa PS1793, PS1796, and PS1797.

The upstream and downstream fragments surrounding the *bla*_OXA-935_ gene were amplified from PS1793 genomic DNA using the following primers: oxa10 5-1-HindIII, oxa10 5-2, oxa10 3-1, and oxa10 3-2-HindIII, of which oxa10 5-2 and oxa10 3-1 contain a 24 bp overlapping linker sequence (*TTCAGCATGCTTGCGGCTCGAGTT*) with which an in-frame deletion of the *bla*_OXA-935_ gene can be generated (Table S9). The resultant upstream and downstream fragments were used as the templates for overlap extension PCR and were amplified with oxa10 5-1-HindIII and oxa10 3-2-HindIII to create a single linear fragment for insertion. The integration proficient vector pEX18HygB was cut with HindIII, and the plasmid and insertion fragment were ligated using the NEB Gibson Assembly Cloning Kit. The resulting vector pEX18HygB-Δ*bla*_OXA-935_ was verified by sequencing at the NuSeq facility at Northwestern University and transformed into E. coli SM10 λpir. Following conjugation and allelic exchange with PS1793, PS1796, and PS1797, whole-genome sequencing was performed on all of the mutant strains to confirm the mutation. Briefly, genomic DNA was isolated from an overnight culture of each parental strain and its corresponding mutant. Sequencing libraries were prepared using a Nextera XT Kit, and sequencing was performed using an Illumina MiSeq instrument and a v2 flow cell, yielding 2 × 251 bp paired end reads. The reads were quality trimmed and aligned to the PS1793 complete genome as previously described. The site of the deletion was examined using Tablet v1.21.02.08 (59).

### RNA extraction and qRT-PCR expression analysis of *bla*_OXA-935_.

Primers and probes were designed for *rpoD* (control gene) and *bla*_OXA-935_ using the Integrated DNA Technologies (IDT) PrimerQuest Tool (amplicon length of 98 bp) (Table S9). Gene blocks for each were generated by IDT as positive controls. To assess the impact of ceftazidime on the expression of *bla*_OXA-935_, P. aeruginosa strains PS1793, PS1796, and PS1797 were grown overnight in triplicate in LB, subcultured 1:50 in the morning, and regrown to mid-log (OD_600_ = 0.5) in the presence and absence of 32 μg/mL ceftazidime (0.5 MIC). To quantify the expression of *bla*_OXA-935_ in strains with inducible expression, PAO1 and PA14 containing pPSV37-*oxa935* were grown overnight in triplicate in LB supplemented with 30 μg/mL of gentamicin, subcultured 1:50 in the morning, and regrown to mid-log (OD_600_ = 0.5) in the presence and absence of 1 mM ITPG. RNA was harvested using the RNeasy Protect Bacteria Minikit (Qiagen, catalog number: 74524). RNA concentrations were measured using a Nanodrop Spectrophotomter, and purities were assessed via formaldehyde agarose gel electrophoresis with a 2.2 M formaldehyde gel (60). cDNA was generated using 2.0 μg of input RNA with SuperScript IV VILO Master Mix (Thermo Fisher Scientific, catalog number: 11756050) containing random primers and oligo(dT). The cDNA samples were stored at −80°C for qRT-PCR analysis. Primer efficiency was assessed, and reference gene (*rpoD*) validation was performed (61, 62). Quantitative RT-PCR (qRT-PCR) was performed in triplicate using TaqMan Fast Advanced Master Mix (Thermo Fisher Scientific, catalog number: 4444556) and analyzed on the Bio-Rad iCycler MyIQ RT-PCR system. No-RT and no-template controls were used. Synthesized gene blocks were used in place of cDNA for positive amplification controls. The expression of *bla*_OXA-935_ was normalized to the expression of the housekeeping gene rpoD. Relative expressions and fold changes were quantified using the comparative C_T_ (2^-ΔΔCT^) method (62). The data were visualized in GraphPad Prism v9.3.1 and analyzed using the Mann-Whitney test.

### Protein production and purification.

The gene sequences of the mature sequence from OXA-14 and OXA-935 were cloned as described above. The plasmids were transformed into E. coli BL21(DE3)(Magic) cells (63), and the transformants were cultured in Terrific Broth (TB) medium supplemented with 200 μg/mL ampicillin and 50 μg/mL kanamycin. The expression of the protein was induced by the addition of 0.5 mM IPTG when the cultures reached OD_600_ = 1.8 to 2.0, and the cultures were further incubated for 14 h at 25°C and 200 rpm (63). The cells were then harvested by centrifugation, resuspended in lysis buffer (50 mM Tris; pH 8.3, 0.5 mM NaCl, 10% glycerol, and 0.1% IGEPAL CA-630), and frozen at −30°C until purification. Frozen suspensions of bacteria were thawed and sonicated at 50% amplitude in a 5 s × 10 s cycle for 20 min at 4°C. The lysate was centrifuged at 39,000 × *g* for 40 min at 4°C, the supernatant was collected, and the protein was purified as previously described, with some modifications (26, 64). The supernatant was loaded onto a His-Trap FF (Ni-NTA) column using a GE Healthcare ÅKTA Pure system in loading buffer (50 mM Na+/Phosphate buffer [pH 7.8], 0.5 M NaCl, 1 mM Tris(2-carboxyethyl) phosphine (TCEP), and 5% glycerol). The column was washed with 10 column volumes (cv) of loading buffer followed by 10 cv of wash buffer (50 mM Na+/phosphate buffer [pH 7.8], 1 M NaCl, 1 mM Tris(2-carboxyethyl) phosphine (TCEP) and 5% glycerol, and 25 mM imidazole) and was eluted with elution buffer (50 mM Na+/phosphate buffer [pH 7.8], 0.5 M NaCl, 1 mM Tris(2-carboxyethyl) phosphine (TCEP) and 5% glycerol, and 1 M imidazole). The protein was loaded onto a Superdex 200 26/600 column and run with loading buffer. The peak fraction was collected, mixed with TEV protease (1:20 protease:protein), and incubated overnight at room temperature to remove the 6×His tag. The cleaved protein was separated from the TEV and the tag by affinity chromatography (Ni-NTA) and dialyzed in crystallization buffer (20 mM K^+^/Na^+^ [pH 7.8]) for 2 h, concentrated to 8 to 9 mg/mL, and either set up for crystallization immediately or flash-frozen and stored at −80°C for further use.

### Crystallization and data collection.

Purified OXA-14 or OXA-935 proteins were set up as 2 μL crystallization drops (1 μL protein:1 μl reservoir solution) in 96-well plates (Corning), using the commercially available Classics II, Anions, and Ammonium Sulfate suites (Qiagen). Diffraction quality crystals of OXA-14 apo-form (PDB code: 7L5R) grew from 0.1 M bicine (pH 9.0), 2.4 M ammonium sulfate, and they were cryoprotected using 2 M lithium sulfate prior to freezing. Crystals of OXA-935 (PDB code: 7L5V) grew from 0.2 M ammonium acetate, 0.1 M Tris (pH 8.5), and 25% PEG 3350. Crystals for the second OXA-935 structure (PDB code: 7N1M) grew from 0.2 M ammonium iodide and 2.2 M ammonium sulfate (Tables S5 and S6), and they were similarly cryoprotected.

The data sets were collected at the beam lines 21ID-D and 21ID-F of the Life Sciences-Collaborative Access Team (LS-CAT) at the Advanced Photon Source (APS), Argonne National Laboratory. Images were indexed, integrated and scaled using HKL-3000 (65).

### Structure solution and refinement.

The OXA-14 and OXA-935 structures were solved by Molecular Replacement with Phaser (65) from the CCP4 Suite (66), using the crystal structure of the OXA-10 (PDB code: 1E3U) as a search model. The initial solutions went through several rounds of refinement in REFMAC v. 5.8.0258 (67) and manual model corrections using Coot (68). The water molecules were automatically generated using ARP/wARP (69), and the ligands were manually fit into electron density maps. The Translation–Libration–Screw (TLS) groups were created by the TLSMD server (70) (http://skuld.bmsc.washington.edu/~tlsmd/), and TLS corrections were applied during the final stages of refinement. MolProbity (71) (http://molprobity.biochem.duke.edu/) was used for monitoring the quality of the model during refinement and for the final validation of the structure. The final model and the diffraction data were deposited into the Protein Data Bank (https://www.rcsb.org/) with the assigned PDB codes: 7L5R (OXA-14), 7L5V (OXA-935), and 7N1M (OXA-935 number 2) (Table S7). Structures were visualized using PyMOL v2.4., Schrodinger, LLC. Composite omit maps were created in CCP4 at the sigma level (72).

### Structural and sequence alignment.

The primary amino acid sequences of OXA-10, OXA-14, and OXA-935 were aligned in Clustal Omega (73). The alignment was used to produce the secondary structure depiction using OXA-935 (PDB code: 7L5V) as the template and the ESPript 3.0 server (74). Additional structure alignments were performed using the POSA (75) and FATCAT (76) servers.

### MICs.

The MICs for the P. aeruginosa strains were determined in triplicate in compliance with the BMD protocol, as outlined by the Clinical and Laboratory Standards Institute, M100-Ed31 (77, 78). The following antibiotics were prepared from commercially available sources and were used to assess MICs: piperacillin-tazobactam (TZP), cefepime (FEP), ceftazidime (CAZ), ciprofloxacin (CIP), meropenem (MEM), gentamicin (GEN), colistin (CST) and aztreonam (ATM). BMD MICs for amikacin (AMK), meropenem-vaborbactam (MVB), imipenem (IPM), imipenem-relebactam (IMR), cefiderocol (FDC), ceftolozane-tazobactam (C/T), and ceftazidime-avibactam (CZA), were determined using commercially available Sensititre Gram Negative Susceptibility Testing Plates (Thermo Fisher Scientific, catalog number: MDRGN2F) per the manufacturer’s protocol. For P. aeruginosa strains containing OXA-containing expression vectors, 0.25 mM to 1 mM IPTG was added to the MIC BMD plate as indicated to induce OXA-β-lactamase expression.

### Kinetic assays.

The enzyme kinetic parameters of the purified OXA-14 and OXA-935 β-lactamases were determined by measuring the initial hydrolysis of pencillin G (λ = 233 nm, Δε = 900 M^−1^cm^−1^), cephalothin (λ = 262 nm, Δε = 7660 M^−1^cm^−1^), ceftazidime (λ = 260 nm, Δε = 7600 M^−1^cm^−1^), cefepime (λ = 260 nm, Δε = 760 M^−1^cm^−1^), meropenem (λ = 298 nm, Δε = 7200 M^−1^cm^−1^), and imipenem (λ = 300 nm, Δε = 9000 M^−1^cm^−1^) in a 200 μL final volume of 100 mM sodium phosphate supplemented with 50 mM sodium bicarbonate (pH 7.0) and 0.01% Triton X-100. The reactions were performed using UV-star 96-well plates (Grenier) at 25°C and were measured on the Tecan Infinite spectrophotometer. The protein concentrations of OXA-14 and OXA-935 were optimized and ranged from 10 to 500 nM. The *k_cat_* (turnover rate) and *K_m_* (Michealis constant) values were obtained via the nonlinear regression of the data using the Michaelis-Menten equation and the plot V/[*E*] versus [S] (in which V is the initial velocity and [*E*] and [S] are the enzyme and substrate concentrations, respectively). All plots and fitted curves were generated using Prism software (GraphPad v.9.1.2). Each experiment was performed in triplicate.

The effects of pH and bicarbonate on OXA-14 and OXA-935 were tested using purified protein (OXA-14 and OXA-935) at 2.5 and 10 nM, respectively, incubated with 50 mM nitrocefin (λ = 490 nm, Δε = 20,500 M^−1^cm^−1^) and prepared in 100 mM sodium phosphate buffer at pH values of 7.0, 7.5, 8.0, 8.5, with or without 50 mM sodium bicarbonate as indicated. Solutions were prepared in a 96-well plate with a total volume of 100 μL. Nitrocefin hydrolysis was measured in clear bottom 96-well plates (Grenier) at 30°C and on the Tecan Safire2 spectrophotometer. Each condition was assayed in triplicate. The slope of the linear regression of the initial velocity of nitrocefin (μM/min) hydrolyzed was determined. The velocity was then determined using the following equation: Velocity (1/s) = ((slope (abs/min)/path length (cm) x Δε) x 1,000,000)/concentration of protein (μM). The velocity was plotted using Prism software (GraphPad v.9.3.1).

### Data availability.

The sequencing and genome assemblies have been deposited to NCBI. The complete genome assembly of PS1793 was deposited into BioProject PRJNA547625 under BioSample SAMN12162657. The genomes of PS1796 and PS1797 are located under the same BioProject with BioSample numbers of SAMN12162658 and SAMN12162659, respectively. The assemblies are available under GenBank accessions GCA_006704595.2, GCA_006704575.1, and GCA_006704565.1. Protein structures were deposited to the Protein Data Bank with the assigned PDB codes: 7L5R (OXA-14), 7L5V (OXA-935), and 7N1M (OXA-935 number 2).
