# Supplementary material for: Functional and Structural Characterization of OXA-935, a Novel OXA-10-Family β-Lactamase from Pseudomonas aeruginosa
Source: Antimicrob Agents Chemother. 2022 Sep 21;66(10):e00985-22. doi: 10.1128/aac.00985-22 (PMC9578422; doi:10.1128/aac.00985-22)
Supplement: Supplemental file 1 — Supplemental material. Download aac.00985-22-s0001.pdf, PDF file, 1.3 MB [file aac.00985-22-s0001.pdf]

Supplementary Information

SUPPLEMENTARY FIGURES

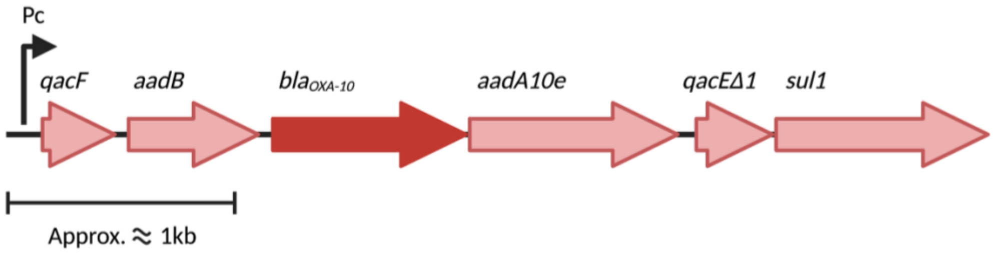

FIGURE S1. Truncated schematic structure of the class I integron, in1697, harboring the OXA-10 β-lactamase driven by the Pc promoter.

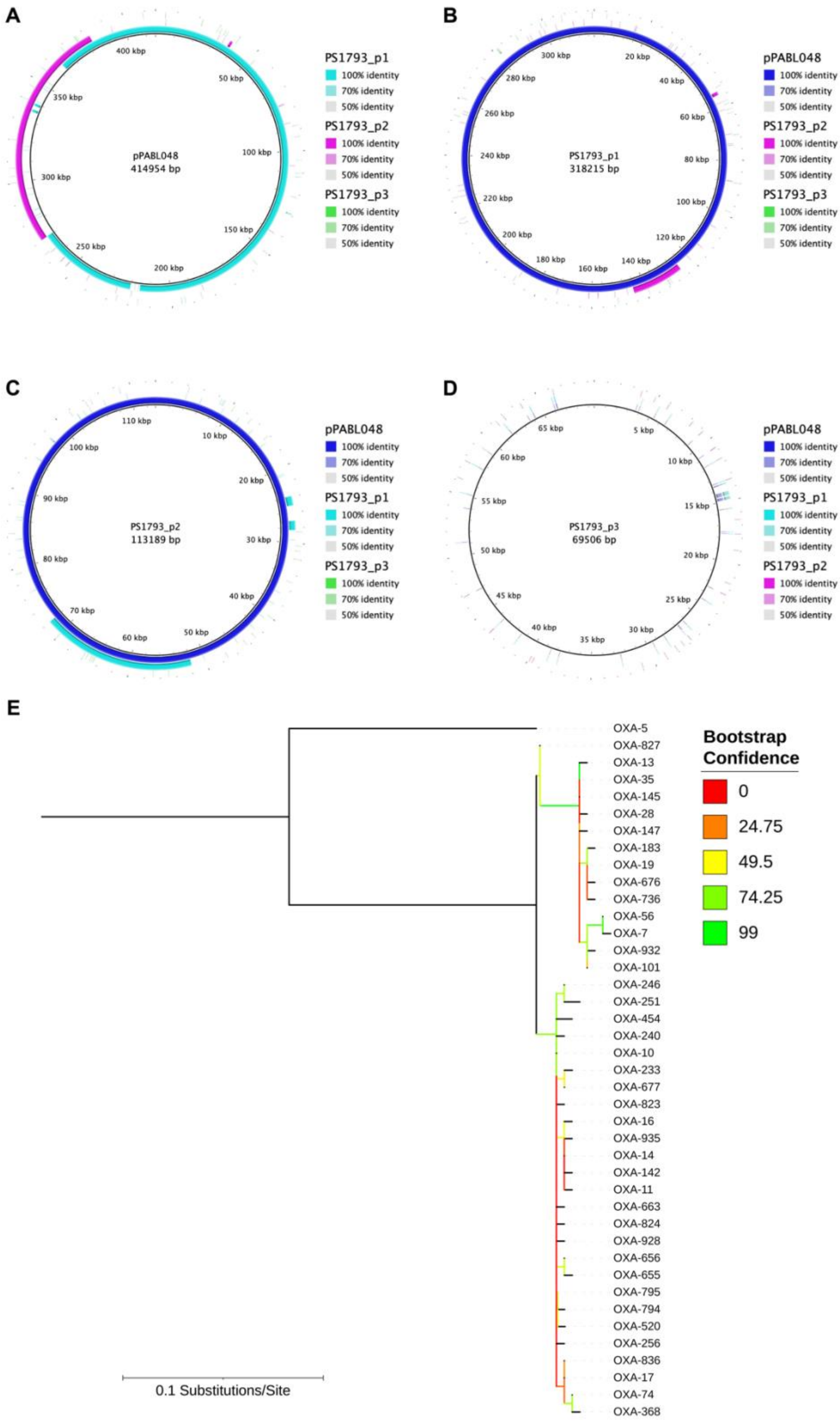

FIGURE S2. Comparison of the plasmids found in *P. aeruginosa* PS1793 with pPABL048 and phylogenetic relationships between OXA-10-like family serine β-lactamases. (A)

Alignment of pPABL048 (blue) with the three plasmids present in PS1793, p1 (teal), p2 (magenta) and p3 (green). **(B, C)** Alignment revealed that two separate plasmids, p1 and p2 shared substantial sequence overlap with pPABL048. PS1793_p1 and p2 also share an overlapping 19 kb region. **(D)** The 69 kb PS1793_p3 plasmid did not share substantial sequence with the previously characterized pPABL048 plasmid. **(E)** Maximum likelihood phylogenetic tree of OXA-10-family serine β-lactamases. Tree is rooted at OXA-5, which was included as an outgroup. Bootstrap confidence is indicated by color. Analysis revealed two major families with OXA-10 and OXA-7 as their earliest-identified members.

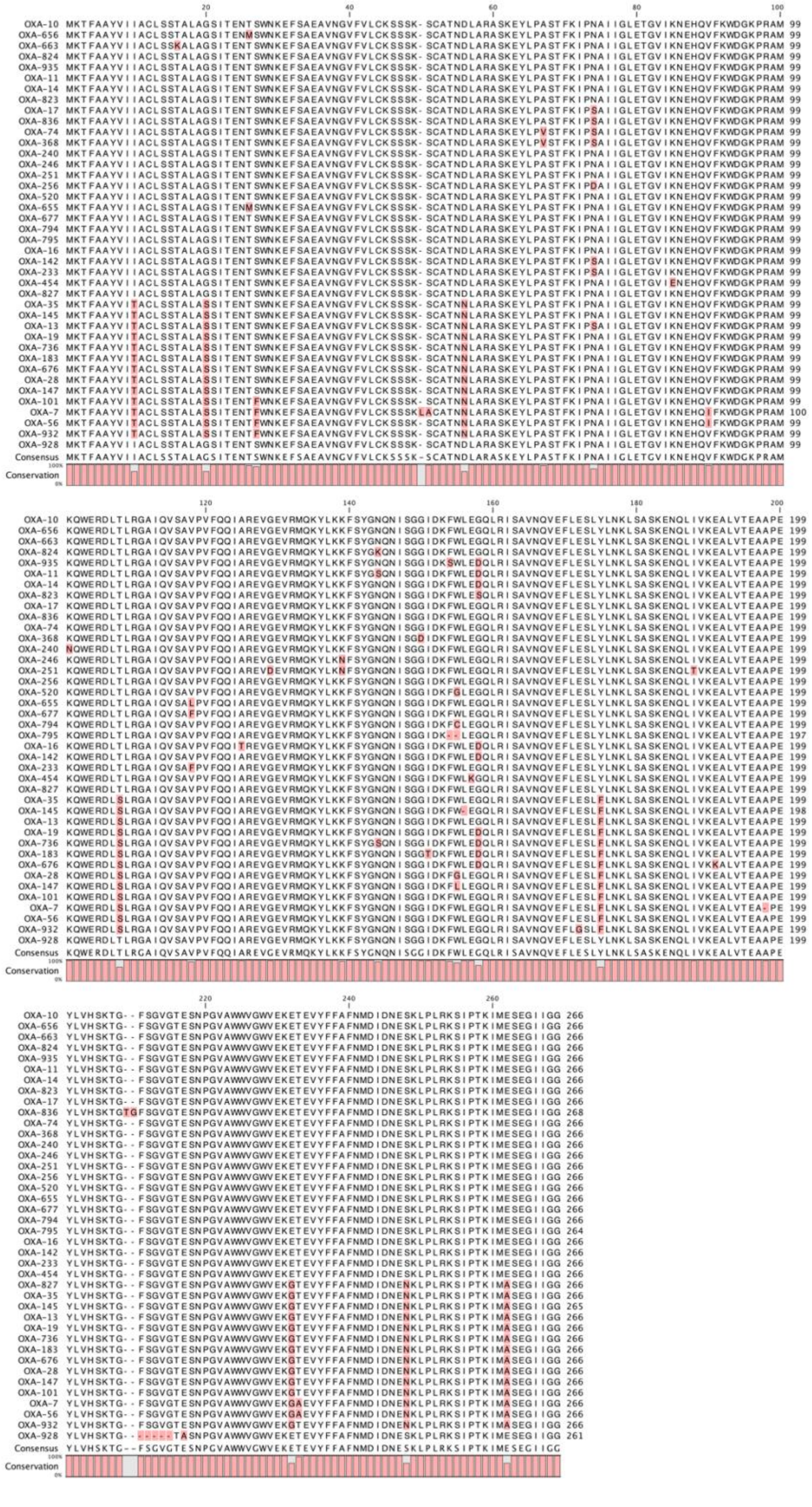

FIGURE S3. Multiple alignment of protein sequences of OXA-10 homologues.

Deviations from the consensus sequence are highlighted in pink.

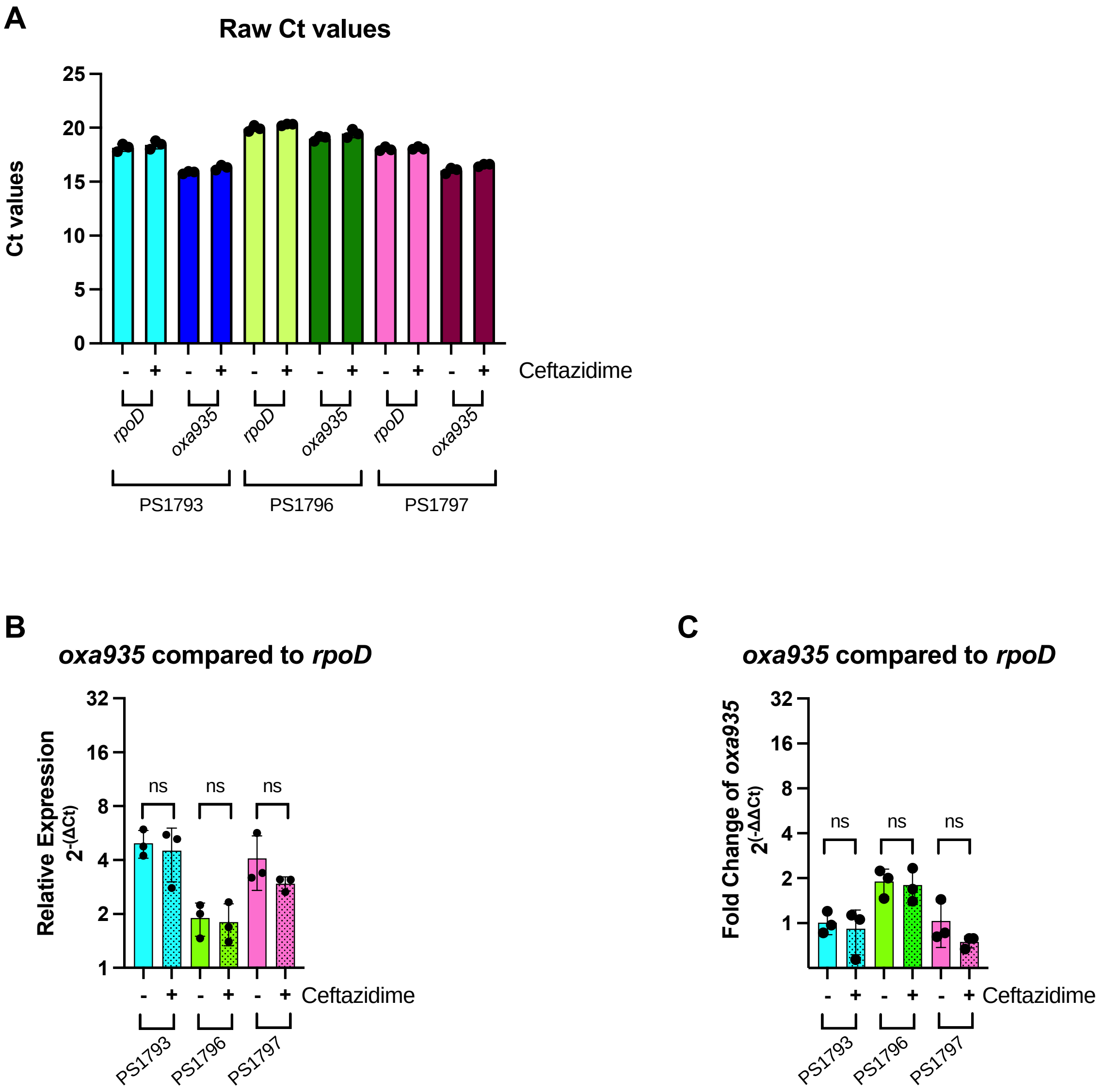

FIGURE S4. Quantitative RT-PCR of *bla*$_{\text{OXA-35}}$ expression in PS1793, 1796, 1797 in the presence of ceftazidime. **(A)** Raw cycle threshold values of expression of the housekeeping gene *rpoD* and *bla*$_{\text{OXA-935}}$ in the absence (-) and presence (+) of 32 µg/mL of ceftazidime (1/2 MIC) in PS1793 (cyan, blue), PS1796 (light green, dark green), and PS1796 (pink, maroon). Relative expression ($2^{-(\Delta Ct)}$) **(B)** and fold change ($2^{(-\Delta\Delta Ct)}$) **(C)** of *bla*$_{\text{OXA-935}}$ compared to *rpoD* control gene in the presence and absence of ceftazidime. ns = not significant by Mann-Whitney.

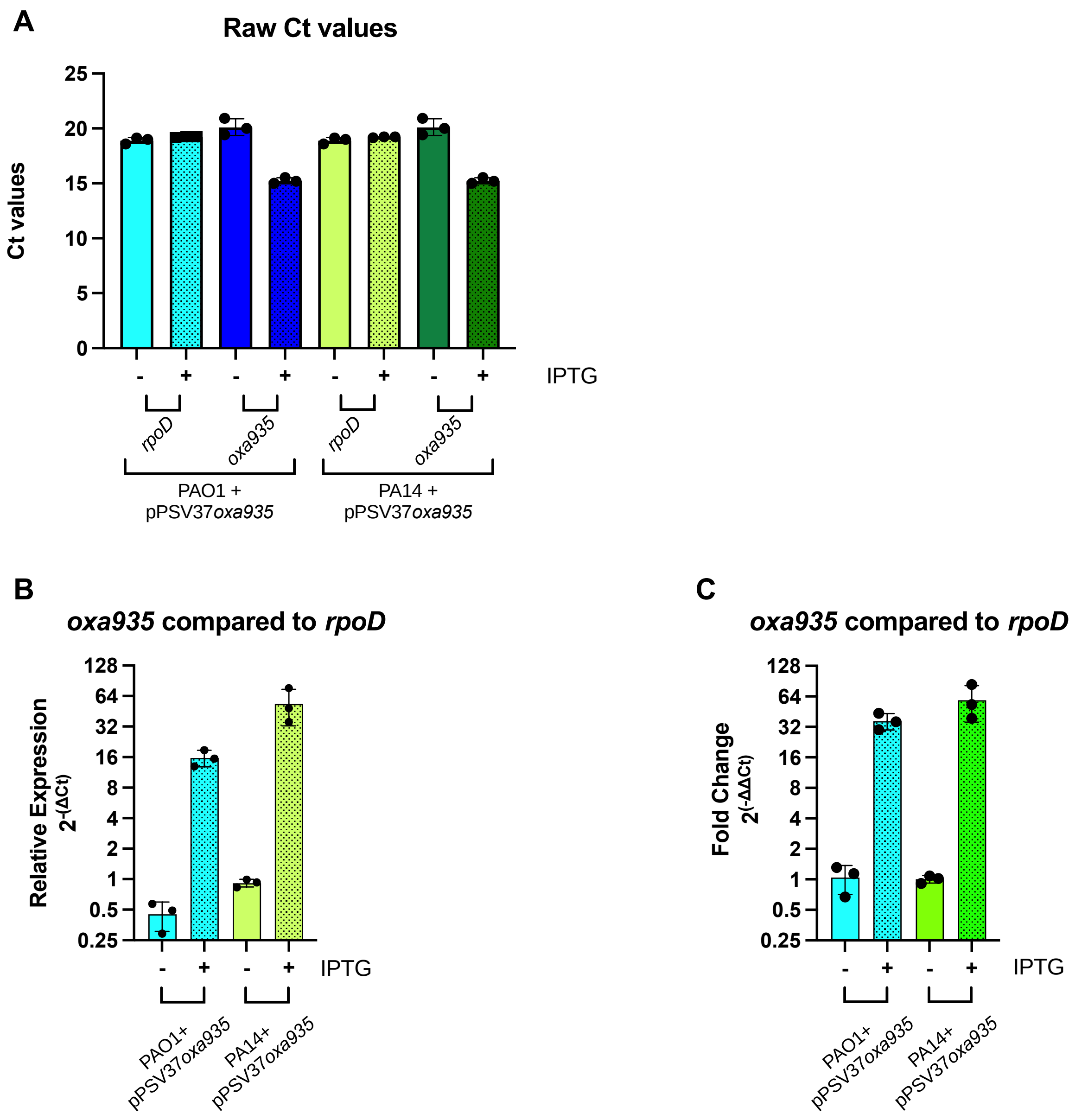

FIGURE S5. Quantitative RT-PCR of *bla*$_{\text{OXA-35}}$ expression in PA01 and PA14 containing pPSV37oxa935. **(A)** Raw cycle threshold values of expression of the housekeeping gene *rpoD* and *bla*$_{\text{OXA-935}}$ in the absence (-) and presence (+) of 1mM of ITPG in PA01 + pPSV37oxa935 (cyan, blue) and PA14 + pPSV37oxa935 (light green, dark green. Relative expression ($2^{-(\Delta Ct)}$) **(B)** and fold change ($2^{(-\Delta\Delta Ct)}$) **(C)** of *bla*$_{\text{OXA-935}}$ compared to *rpoD* control gene in the presence and absence of 1mM ITPG.

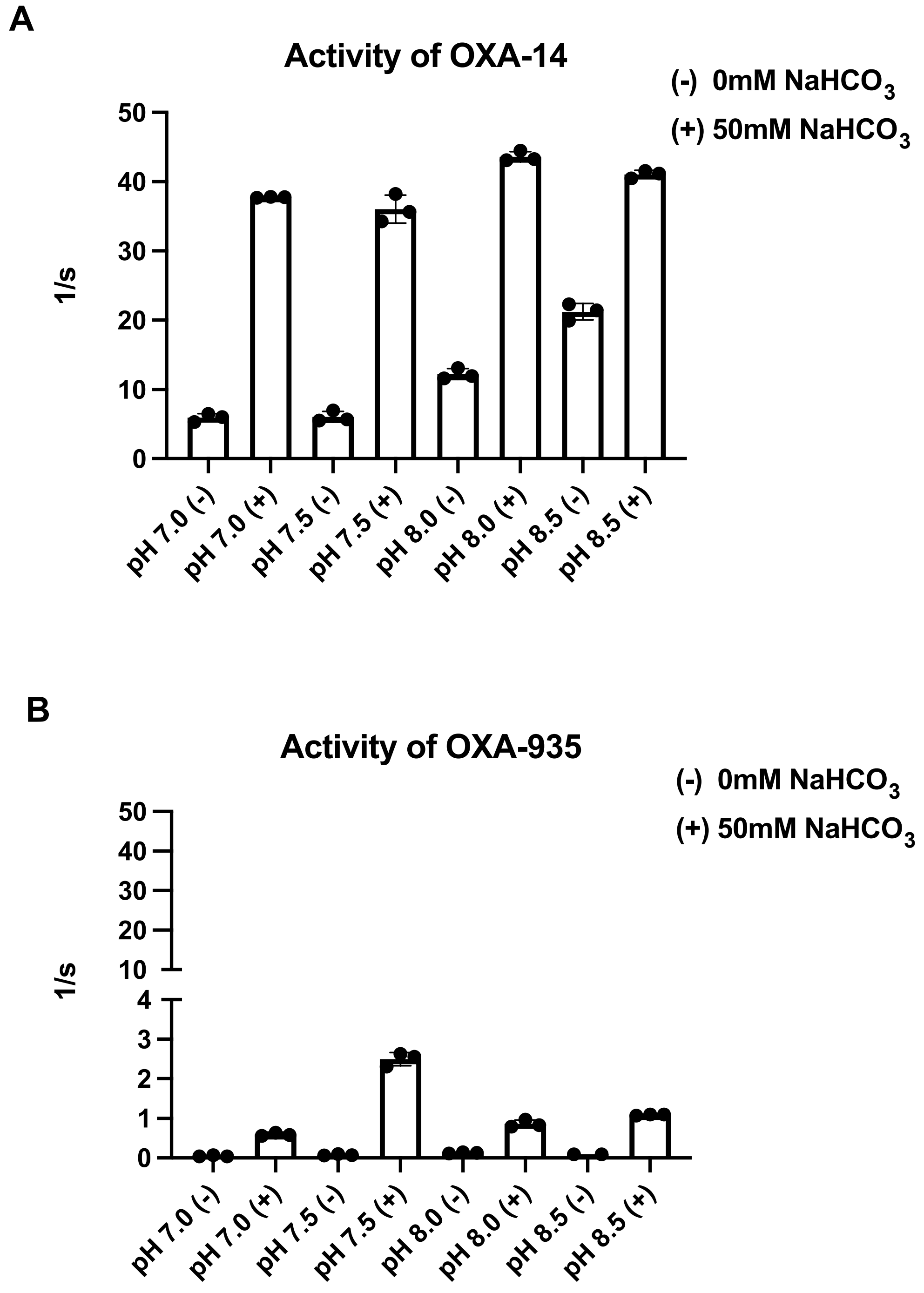

FIGURE S6. High pH and $NaHCO_3$ stimulate the activity of OXA-14. The plot represents the specific activity (1/s) of **(A)** OXA-14 and **(B)** OXA-935 using nitrocefin hydrolysis as a reporter in the absence (-) or presence (+) of 50 mM $NaHCO_3$ at different pH values. Experiments were performed in triplicate. Means and standard deviations are presented.

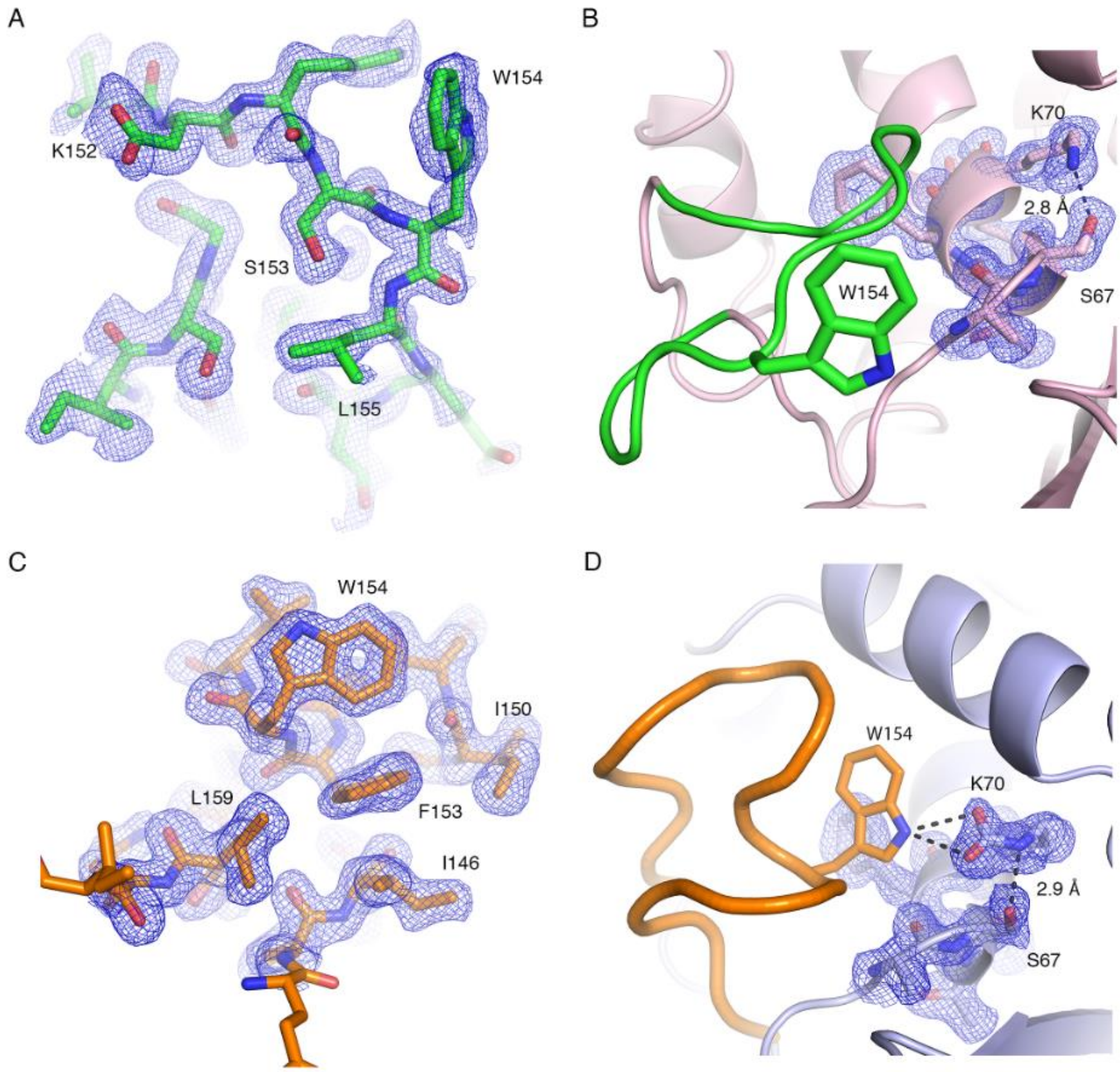

FIGURE S7. Confirmation of the Ω-loop and position of the active site residues in OXA-925 and OXA-14. Composite omit maps represented as blue mesh surrounding the (**A**) Ω-loop and (**B**) active site residues in OXA-935 and the (**C**) Ω-loop and (**D**) active site residues in OXA-14. Residues are shown as sticks with oxygens in red, nitrogens in blue, carbons in green (Ω-loop, OXA-935), pink (active site, OXA-935), orange (Ω-loop, OXA-14), and pale blue (active site, OXA-14), respectively. Selected residues are labeled and hydrogen bond interactions are shown as dashed lines.

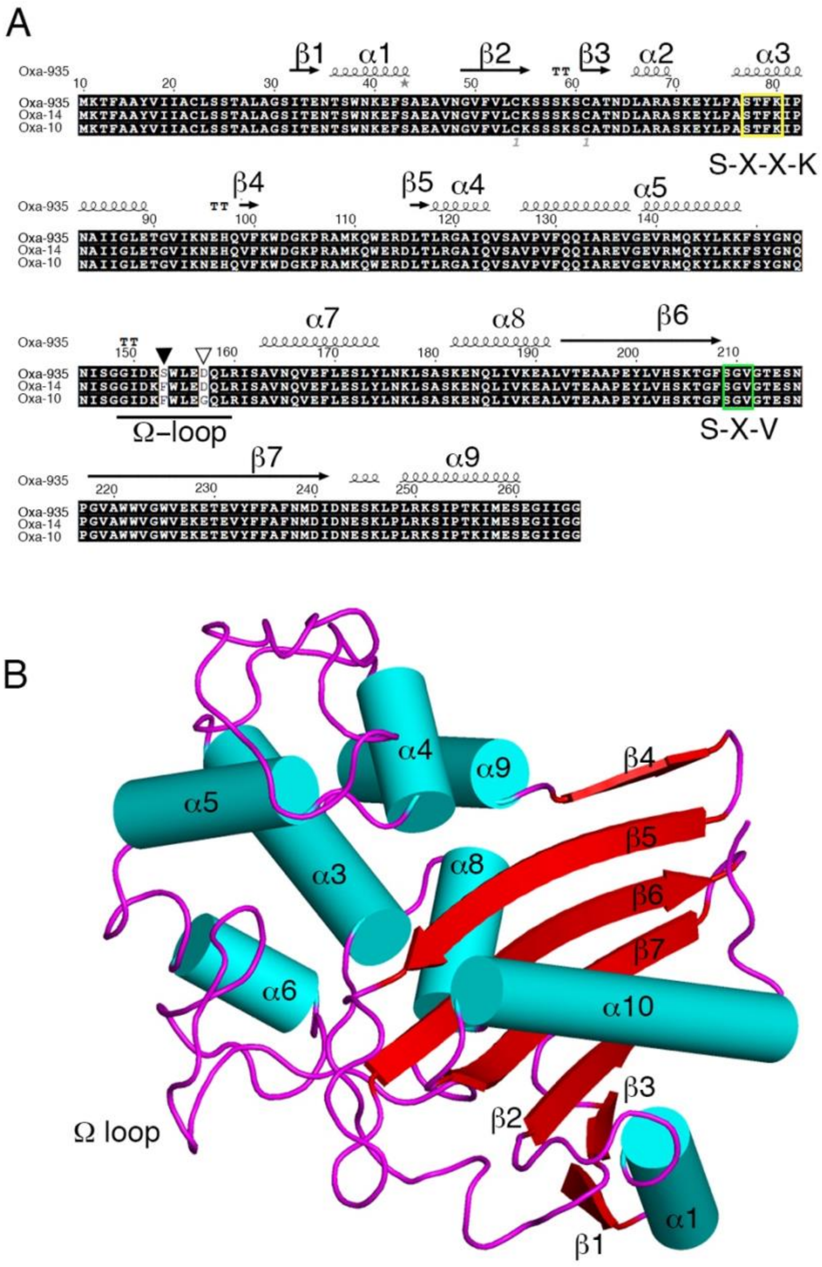

FIGURE S8. Sequence alignment and structural elements of OXA-10-variants. (A) Sequence alignment of OXA-935, OXA-14, and OXA-10 where the closed arrowhead highlights the variation at position 153 and the open arrowhead highlights the variation at position 157. Secondary structure elements shown on top of the aligned sequences are based on the OXA-935 structure with α-helices represented as spirals and β-strands as arrows. The catalytic residues, S-X-X-K are highlighted in yellow and S-X-V in green. **(B)** Cartoon representation of the structure of OXA-935, where loops are colored in pink, α-helices are shown as teal cylinders and β-strands as red arrows.

Table S1: Amino acid changes PS1793, PS1796 and PS1797 in genes implicated in β-lactam resistance

Gene	PAO1 identifier	PA14 identifier	Function	Δaa from PAO1*	Δaa from PA14*
ampC[a]	PA4110	PA14_10790	beta-lactamase	G1D, T79A, V179L, G365A[a]	T29A, R129Q[a]
ampD	PA4522	PA14_58670	zinc protease	**early frameshift**	**early frameshift**
ampR	PA4109	PA14_10800	transcriptional regulator	E114A, G183E, M288R	-
ampDh2	PA5485	PA14_72400	zinc protease	-	-
ampDh3	PA0807	PA14_53820	zinc protease	**A208V**	**A208V**
dacB (PBP-4)	PA3047	PA14_24690	pencillin binding protein 4, endopeptidase, carboxypeptidase	-	H156Q
creB (blrA)	PA0463	PA14_06060	two-component regulator	**A130T**	V39A, **A130T**
creC (blrB)	PA0464	PA14_06070	two-component sensor	G157A	-
parR	PA1799	PA14_41260	two-component response regulator	L153R, S170N, **G232D**	**G232D**
parS	PA1798	PA14_41270	two-component sensor	H398R	-
ftsI (PBP-3)	PA4418	PA14_57425	penicillin-binding protein 3, transpeptidase	-	-
mexR	PA0424	PA14_05520	multidrug resistance operon repressor	V126E	-
nalC	PA3721	PA14_16280	transcriptional regulator	**Δ12-15**, G71E, A145V, S209R	**Δ12-15**
nalD	PA3574	PA14_18080	transcriptional regulator	-	-
mexA	PA0425	PA14_05530	RND multidrug efflux membrane fusion protein	-	-
mexB	PA0426	PA14_05540	RND multidrug efflux transporter	-	-
oprM	PA0427	PA14_05550	major intrinsic multiple antibiotic resistance outer membrane protein	-	-
nfxB	PA4600	PA14_60860	transcriptional regulator	-	H21R, G56D
mexC	PA4599	PA14_60850	RND multidrug efflux membrane fusion protein	H310R, S330A, A378T, P383S, A384V	S47P, Q76R
mexD	PA4598	PA14_60830	RND multidrug efflux transporter	E257Q, S845A	S87T, T155A, D669N, G685S, V703I, A915S, R1031K, T1040S
oprJ	PA4597	PA14_60820	multidrug efflux outer membrane protein	M69V, **T376S**	G68D, **T376S**
mexS	PA2491	PA14_32420	mexT suppressor	D249N	-
mexT	PA2492[b]	PA14_32410[c]	transcriptional regulator	n/a	-
cmrA	PA2047	PA14_38040	chloramphenicol resistance activator	**E61D**	**E61D**

mvaT	PA4315	PA14_56070	transcriptional regulator	-	-
PA3271	PA3271	PA14_21700	probable two-component sensor	A212V, **Y942C**	**Y942C**
mexE	PA2493	PA14_32400	RND multidrug efflux membrane fusion protein	**S8F**	**S8F**
mexF	PA2494	PA14_32390	RND multidrug efflux transporter	-	-
oprN	PA2495	PA14_32380	multidrug efflux outer membrane protein	S13P	-
mexZ	PA2020	PA14_38380	transcriptional regulator	**L163P**	R138L, **L163P**, S186N
amgS	PA5199	PA14_68680	probable two-component system	I260V	-
mexX	PA2019	PA14_38395	RND multidrug efflux membrane fusion protein	A30T, K329Q, L331V, W358R	-
mexY	PA2018	PA14_38410	RND multidrug efflux transporter	I536V, T542A, G589A, Q840E, N1036T	Q175E, Q421E, T429A, H908L, R1039Q
oprD	PA0958	PA14_51880	multidrug efflux outer membrane protein	T103S, K115T, F170L, E185Q, P186G, V189T, **S278P**, R310E, A315G, G425A	**S278P**

*Mutations unique to PS1793, PS1796 and PS1797 in comparison to both PAO1 and PA14 are highlighted in bold
[a]Nomenclature for numbering amino acids in AmpC begins after cleavage of the 26 amino acid signal peptide (e.g. G27 is now relabeled G1)
[b]PA2492 (PAO1 mexT) is inactive because of an early insertion leading to a frameshift. Therefore, comparison with PS1793 mexT was not undertaken.
[c] mexT of PS1793 was originally annotated as a pseudogene by NCBI. However, further examination of the sequence revealed that mexT of PS1793 was identical to PA14_32410.

TABLE S2 Alignment of PS1796 and PS1797 to the PS1793 Complete Genome

Isolate	PS1793 Chromosome		PS1793 p1		PS1793 p2		PS1793 p3	
	Percent Alignment[a]	SNVs	Percent Alignment[a]	SNVs	Percent Alignment[a]	SNVs	Percent Alignment[a]	SNVs
PS1796	99.02	0	99.86	0	99.84	0	99.03	0
PS1797	98.87	1	99.96	0	99.65	0	98.48	0

[a]Percentage of total length of covered by aligned sequences with a minimum depth cutoff of 5 reads.

TABLE S3 Identification of Any OXA-10 Family Gene in *Pseudomonas* Genomes by Species

Species	Number of Genomes[ab]
Pseudomonas aeruginosa	196
Pseudomonas asiatica	1
Pseudomonas oleovorans	1
Pseudomonas putida	2
Pseudomonas sp.	2
Pseudomonas stutzeri	8
Pseudomonas xanthomarina	1

[a]Based off BLAST search of 9799 *Pseudomonas* genomes with 90% sequence identity and coverage cutoffs

[b]Only species with at least one OXA-10 family gene identified are displayed

TABLE S4 OXA-10 Family Genes in *Pseudomonas* by Species and MLST

Gene	Number of Genomes[a]	Count by Species and MLST
bla$_{OXA-10}$	118	*Pseudomonas aeruginosa*: 106 (ST233 - 1, ST234 - 7, ST235 - 11, ST244 - 8, ST270 - 1, ST298 - 15, ST308 - 2, ST309 - 5, ST316 - 5, ST357 - 12, ST532 - 1, ST664 - 6, ST708 - 2, ST767 - 1, ST773 - 3, ST1047 - 2, ST1076 - 4, ST1129 - 1, ST1419 - 2, ST2592 - 1, ST2712 - 1, ST3014 - 1, Unknown ST - 14), *Pseudomonas stuzeri*: 8, *Pseudomonas asiatica:* 1, *Pseudomonas olevorans*: 1, *Pseudomonas xanthomarina*: 1, *Pseudomonas sp.*: 1
bla$_{OXA-56}$	46	*Pseudomonas aeruginosa*: 46 (ST235 - 1, ST277 - 44, Unknown ST - 1)
bla$_{OXA-17}$	10	*Pseudomonas aeruginosa*: 10 (ST235 - 7, ST446 - 2, ST639 - 1)
bla$_{OXA-101}$	5	*Pseudomonas aeruginosa*: 4 (ST111 - 2, Unknown ST - 2), *Pseudomonas asiatica*: 1
bla$_{OXA-19}$	4	*Pseudomonas aeruginosa*: 3 (ST235 - 2, ST549 - 1), *Pseudomonas putida*: 1 (Unknown ST - 1)
bla$_{OXA-35}$	4	*Pseudomonas aeruginosa*: 3 (ST235 - 2, ST348 - 1), *Pseudomonas putida*: 1 (ST70 - 1)
bla$_{OXA-935}$	3	*Pseudomonas aeruginosa*: 3 (ST298 - 3)
bla$_{OXA-74}$	2	*Pseudomonas aeruginosa*: 2 (ST235 - 2)
bla$_{OXA-14}$	1	*Pseudomonas aeruginosa:* 1 (ST235 - 1)
bla$_{OXA-28}$	1	*Pseudomonas aeruginosa*: 1 (ST235 - 1)
bla$_{OXA-246}$	1	*Pseudomonas* sp.: 1

[a]Based off BLAST search of 9799 *Pseudomonas* genomes with 100% sequence identity and coverage cutoffs

TABLE S5 Crystallization Information

	**OXA-14**	**OXA-935 (monoclinic)**	**OXA-935 (orthorhombic)**
PBD code	75LR	7L5V	7N1M
Method	Vapor diffusion, sitting drop	Vapor diffusion, sitting drop	Vapor diffusion, sitting drop
Plate type	Corning 96-well plate for crystallization	Corning 96-well plate for crystallization	Corning 96-well plate for crystallization
Temperature (˚C)	20	20	20
Protein Concentration (mg ml^{-1})	8.0	6.0	6.0
Protein solution	0.05 M Sodium phosphate, pH 7.8	0.05 M Sodium phosphate, pH 7.8	0.05 M Sodium phosphate, pH 7.8
Reservoir solution	0.1 M Bicine pH 9.0, 2.4 M Ammonium sulfate	0.2 M Ammonium acetate, 0.1 M Tris pH 8.5, 25% (w/v) PEG 3350	0.2 M Ammonium iodide, 2.2 M Ammonium sulfate
Volume and ratio of drop	2 µL (1:1)	2 µL (1:1)	2 µL (1:1)
Volume of reservoir (µL)	85 µL	85 µL	85 µL

TABLE S6 Data collection and processing

	**OXA-14**	**OXA-935**	**OXA-935**
Diffraction source	21ID-F	21ID-D	21ID-D
Wavelength (Å)	0.97872	1.12713	1.12713
Temperature (K)	100.0	100.0	100.0
Detector	Rayonix MX-300	Dectris Eiger 9M	Dectris Eiger 9M
Space Group	$P2_12_12_1$	$P2_1$	$P2_12_12_1$
a,b,c (Å)	48.81, 96.35, 125.14	46.07, 75.04, 82.75	47.68, 91.38, 125.79
α, β, γ (˚)	90.00, 90.00, 90.00	90.00, 91.93, 90.00	90.00, 90.00, 90.00
Resolution range (Å)	30.00 – 1.65 (1.68 – 1.65)[a]	30.00 – 1.30 (1.32 – 1.30)	30.00 – 1.95 (1.98 – 1.95)
No. of unique reflections	71,761 (3,530)	126,515 (5,121)	40,444 (2,011)
Completeness (%)	100.0 (100.0)	91.2 (74.0)	99.8 (100.0)
Multiplicity	6.0 (6.1)	5.6 (4.9)	11.1 (10.9)
($I/\sigma(I)$)	27.0 (2.4)	20.6 (3.2)	19.4 (2.0)
$R_{p.i.m.}$ [a]	0.027 (0.365)	0.033 (0.247)	0.046 (0.544)
$CC_{1/2}$ [b]	0.999 (0.800)	0.997 (0.854)	0.994 (0.538)
Overall B factor from Wilson Plot (Å^2)	21.1	10.6	33.4

Notes: values in parenthesis are for the outer shell

[a] Estimated Rp.i.m as defined by Weiss, 2001 (1).

[b] Person's correlation coefficient as defined by Karplus and Diedrichs, 2012 (2).

TABLE S7 Structure refinement

	**OXA-14**	**OXA-935**	**OXA-935**
Resolution range (Å)	29.75 – 1.65 (1.69 – 1.65)	23.95 – 1.30 (1.33 – 1.30)	29.77 - 1.96 (2.00 - 1.96)
Completeness (%)	99.7 (97.6)	91.3 (79.4)	99.4 (95.7)
No. of reflections, working set	68,142 (4,889)	119,981 (7,667)	38,401 (2,681)
No. of reflections, test set	3,543 (237)	6,069 (390)	1,983 (135)
Final R_{work}	0.164 (0.245)	0.149 (0.232)	0.187 (0.296)
Final R_{free}	0.190 (0.259)	0.170 (0.251)	0.231 (0.302)
No. of non-H atoms			
Protein	3,917	4,092	3,798
Water	505	725	170
Ligand	128	0	110
R.m.s. deviations			
Bonds (Å)	0.006	0.005	0.006
Angles (˚)	1.336	1.287	1.415
Average B factors (Å^2)	25.9	15.8	47.0
Ramachandran plot[c]			
Favored regions (%)	97.0	99.0	100.0
Additionally allowed (%)	3.0	1.0	0.0
Outliers (%)	0.0	0.0	0.0

[c]Validation was done using MolProbity (3).

TABLE S8 Bacterial strains used in this study

Species	Strain ID	Relevant Characteristics	Reference
E. coli	TOP-10	F- mcrA Δ(mrr-hsdRMS-mcrBC) φ80lacZΔM15 ΔlacX74 nupG recA1 araD139 Δ(ara-leu)7697 galE15 galK16 rpsL(Str^R) endA1 λ-	Invitrogen
E. coli	S17-1 λpir	Sm^R; pro, thi, $hsdR^-M^+$, RP4-2-Tc:Mu;Km:Tn7 λpir	Simon (1983) (4)
E. coli	SM10 λpir	Km^R, thi-1 thr leu tonA lacY supE recA::RP4-2-Tc::Mu λpir	Simon (1983)
E. coli	BL21(DE3)(pMagic)	Km^R, F- ompT gal dcm lon hsdSB(rB^-mB^-) λ(DE3[lacI lacUV5-T7p07 ind1 sam7 nin5]) $[malB^+]_{K-12(}λ^S$), derivative of BL21(DE3) expressing pMagic plasmid which encodes three rare-triplet tRNAs (AGG for Arg, AGA for Arg, and ATA for Ile)	Wu (2000) (5)
P. aeruginosa	PA14	human wound isolate	Rahme (1995) (6)
P. aeruginosa	PAO1	human wound isolate	Holloway (1955) (7)
P. aeruginosa	PABL048	human bacteremia isolate, Assembly: GCA_003411785.2	Scheetz (2009) (8)
P. aeruginosa	PS1793	human respiratory isolate, Assembly: GCA_006704595.1	Pincus (2020) (9)
P. aeruginosa	PS1796	human respiratory isolate, Assembly: GCA_006704575.1	Pincus (2020)
P. aeruginosa	PS1797	human respiratory isolate, Assembly: GCA_006704565.1	Pincus (2020)
P. aeruginosa	PS1793Δ*oxa935*	Clean deletion of *oxa935* (Δaa5-262)	This study
P. aeruginosa	PS1796Δ*oxa935*	Clean deletion of *oxa935* (Δaa5-262)	This study
P. aeruginosa	PS1797Δ*oxa935*	Clean deletion of *oxa935* (Δaa5-262)	This study

TABLE S9 Plasmids and primers

**Plasmid & Primers**	**Relevant Characteristics or Sequence 5' – 3'**	**Reference**
pFLP-hyg	Hyg^R; p15a ts ori, expresses FLP recombinase	Huang (2014) (10)
pEX18Ap	Amp^R; allelic exchange vector for making unmarked deletions in *P. aeruginosa*	Hoang (1998) (11)
pEX18HygB	Hyg^R; allelic exchange vector, derivative of pEXT18Ap	This study
pEX18HygB-Δbla_{oxa935}	Hyg^R; allelic exchange vector for making unmarked deletion of *oxa935* (Δ5-262)	This study
pMCSG53	Ap^R/Cb^R; ColE1/pBR322/pUC ori+, N-term TEV cleavage site, N-term His_6 tag	Eschenfeldt (2013)(12)
pMCSG53-*oxa14* FL	Ap^R/Cb^R; full length version of *oxa14* cloned in frame into the SspI site	This study
pMCSG53-*oxa935* FL	Ap^R/Cb^R; full length version of *oxa935* cloned in frame into the SspI site	This study
pMCSG53-*oxa14* trunc	Ap^R/Cb^R; *oxa14* Δaa1-20 (signal sequence) cloned in frame into the SspI site	This study
pMCSG53-*oxa935* trunc	Ap^R/Cb^R; *oxa935* Δaa1-20 (signal sequence) cloned in frame into the SspI site	This study
pPSV37	Gm^R, ColE1/pMB1/pBR322/pUC ori+, *lacI*^{q}, and the *lac*UV5 promoter, derivative of pPSV35	Lee (2010)
pPSV37-*oxa10*	Gm^R, full length *oxa10* cloned into HindIII site in MCS of pPSV37	This study
pPSV37-*oxa14*	Gm^R, full length *oxa14* cloned into HindIII site in MCS of pPSV37	This study
pPSV37-*oxa935*	Gm^R, full length *oxa935* cloned into HindIII site in MCS of pPSV37	This study
TT113	ATTAGCTTCAAAAGCGCTCTCGTGAGTTTTCGTTCCACTGA	This study
TT114	TGCAGCGAATTGGGGATCTTGAAAACCTCTGACACATGCAG	This study
TT115	AGAGCGCTTTTGAAGCTAATCCCGACGCACTTTGCAGATCT	This study
TT116	AAGATCCCCAATTCGCTGCAGAGTTTTCGCCCCGAAGAACG	This study
TT117	TGCAGCGAATTGGGGATCTTG	This study
TT118	AGATTCTTCGCCTTGGTAGCC	This study
TT119	CTTGTTTTGCAAACTTTTTGA	This study
TT120	GGTTTCATCAGCCATCCGCTT	This study
TT121	ACTCTCGCATGGGGAGACCCC	This study
TT122	AGCAAAAGGCCAGCAAAAGGC	This study
TT123	CCGCTCATGAGACAATAACCC	This study
TT124	ACTTCGAGCGGAGGCATCCGG	This study
TT125	CCCCAGCACTCGTCCGAGGGC	This study
oxa10 5 up	TGGCGTTGCGTATGCTCACAG	This study
oxa10 3 down	CGAGTCCCGACACCAGACTGCAT	This study
oxa10 5-1-HindIII	GTAAAACGACGGCCAGTGCCAGTCTCTCGCGCTCAAGT	This study
oxa10 5-2	*AACTCGAGCCGCAAGCATGCTGAA*AAATGTTTTCATGGCACCTTG	This study
oxa10 3-1	*TTCAGCATGCTTGCGGCTCGAGTT*ATTGGTGGCTAAAACAAAGTT	This study
oxa10 3-2-HindIII	GTCGACCTGCAGGCATGCAGGCTTCATGCTTCACG	This study
pPSV37_OXA_F_Gibs	CGACCTGCAGGCATGCAATGAAAACATTTGCCGCATATG	This study
pPSV37_OXA_R_Gibs	CCAAGGGGTTATGCTAATTAGCCACCAATGATGCCC	This study
pPSV37_OXA_trunc_F_Gibs	CGACCTGCAGGCATGCAATGTCAATTACAGAAAATACGTCTTG	This study
SeqFwPr PSV37	GCGATCAAAAAACCCCTCAA	This study
SeqRevPr PSV37	CAGGAAACAGCTATGACCAT	This study
rpoD forward	GGGCGAAGAAGGAAATGGT	This study
rpoD reverse	CTGGATCAGGTCGAGGAATTG	This study

rpoD probe	TCCATCGCCAAGAAGTACACCAACC	This study
blaOXA935 forward	TGTCTTTCGAGTACGGCATTAG	This study
blaOXA935 reverse	CAAAGCACGAAGACACCATTG	This study
blaOXA935 probe	AAAGAGTTCTCTGCCGAAGCCGT	This study

REFERENCES

1. Weiss MS. 2001. Global indicators of X-ray data quality. Journal of Applied Crystallography 34:130-135.
2. Karplus PA, Diederichs K. 2012. Linking crystallographic model and data quality. Science 336:1030-3.
3. Chen VB, Arendall WB, 3rd, Headd JJ, Keedy DA, Immormino RM, Kapral GJ, Murray LW, Richardson JS, Richardson DC. 2010. MolProbity: all-atom structure validation for macromolecular crystallography. Acta Crystallogr D Biol Crystallogr 66:12-21.
4. Simon R, Priefer U, Pühler A. 1983. A Broad Host Range Mobilization System for In Vivo Genetic Engineering: Transposon Mutagenesis in Gram Negative Bacteria. Bio/Technology 1:784-791.
5. Wu N, Christendat D, Dharamsi A, Pai EF. 2000. Purification, crystallization and preliminary X-ray study of orotidine 5'-monophosphate decarboxylase. Acta Crystallogr D Biol Crystallogr 56:912-4.
6. Rahme LG, Stevens EJ, Wolfort SF, Shao J, Tompkins RG, Ausubel FM. 1995. Common virulence factors for bacterial pathogenicity in plants and animals. Science 268:1899-902.
7. Holloway BW. 1955. Genetic recombination in *Pseudomonas aeruginosa*. J Gen Microbiol 13:572-81.
8. Scheetz MH, Hoffman M, Bolon MK, Schulert G, Estrellado W, Baraboutis IG, Sriram P, Dinh M, Owens LK, Hauser AR. 2009. Morbidity associated with *Pseudomonas aeruginosa* bloodstream infections. Diagnostic Microbiology and Infectious Disease 64:311-319.
9. Pincus NB, Bachta KER, Ozer EA, Allen JP, Pura ON, Qi C, Rhodes NJ, Marty FM, Pandit A, Mekalanos JJ, Oliver A, Hauser AR. 2020. Long-term Persistence of an Extensively Drug-Resistant Subclade of Globally Distributed *Pseudomonas*

aeruginosa Clonal Complex 446 in an Academic Medical Center. Clin Infect Dis 71:1524-1531.

10. Huang TW, Lam I, Chang HY, Tsai SF, Palsson BO, Charusanti P. 2014. Capsule deletion via a lambda-Red knockout system perturbs biofilm formation and fimbriae expression in *Klebsiella pneumoniae* MGH 78578. BMC Res Notes 7:13.
11. Hoang TT, Karkhoff-Schweizer RR, Kutchma AJ, Schweizer HP. 1998. A broad-host-range Flp-FRT recombination system for site-specific excision of chromosomally-located DNA sequences: application for isolation of unmarked *Pseudomonas aeruginosa* mutants. Gene 212:77-86.
12. Eschenfeldt WH, Makowska-Grzyska M, Stols L, Donnelly MI, Jedrzejczak R, Joachimiak A. 2013. New LIC vectors for production of proteins from genes containing rare codons. J Struct Funct Genomics 14:135-44.
